# Side- and Disease-Dependent Changes in Human Aortic Valve Cell Population and Transcriptomic Heterogeneity Determined by Single-Cell RNA Sequencing

**DOI:** 10.3390/genes15121623

**Published:** 2024-12-19

**Authors:** Nicolas Villa-Roel, Christian Park, Aitor Andueza, Kyung In Baek, Ally Su, Mark C. Blaser, Bradley G. Leshnower, Ajit Yoganathan, Elena Aikawa, Hanjoong Jo

**Affiliations:** 1 Wallace H. Coulter Department of Biomedical Engineering, Emory University and Georgia Institute of Technology, 1760 Haygood Drive, Health Sciences Research Bldg E170, Atlanta, GA 30322, USAcpark373@gatech.edu (C.P.); andueza.aitor@gmail.com (A.A.); kbaek8@emory.edu (K.I.B.); ally.jane.su@emory.edu (A.S.); ajit.yoganathan@bme.gatech.edu (A.Y.); 2 Center for Interdisciplinary Cardiovascular Sciences, Cardiovascular Division, Department of Medicine, Brigham and Women’s Hospital, Harvard Medical School, Boston, MA 02115, USA; mblaser@bwh.harvard.edu (M.C.B.); eaikawa@bwh.harvard.edu (E.A.); 3 Division of Cardiothoracic Surgery, Emory University, Atlanta, GA 30322, USA; bleshno@emory.edu; 4 Department of Medicine, Emory University, Atlanta, GA 30322, USA

**Keywords:** calcific aortic valve disease, aortic sclerosis, single-cell RNA sequencing, human aortic valves, aortid stenosis, transcriptomics, AP-1 related transcription factors, inflammation, endothelial-to-mesenchymal transition, valve endothelial cells, valve interstitial cells

## Abstract

Background: Calcific aortic valve disease (CAVD) is a highly prevalent disease, especially in the elderly population, but there are no effective drug therapies other than aortic valve repair or replacement. CAVD develops preferentially on the fibrosa side, while the ventricularis side remains relatively spared through unknown mechanisms. We hypothesized that the fibrosa is prone to the disease due to side-dependent differences in transcriptomic patterns and cell phenotypes. Methods: To test this hypothesis, we performed single-cell RNA sequencing using a new method to collect endothelial-enriched samples independently from the fibrosa and ventricularis sides of freshly obtained human aortic valve leaflets from five donors, ranging from non-diseased to fibrocalcific stages. Results: From the 82,356 aortic valve cells analyzed, we found 27 cell clusters, including seven valvular endothelial cell (VEC), nine valvular interstitial cell (VIC), and seven immune, three transitional, and one stromal cell population. We identified several side-dependent VEC subtypes with unique gene expression patterns. Homeostatic VIC clusters were abundant in non-diseased tissues, while VICs enriched with fibrocalcific genes and pathways were more prevalent in diseased leaflets. Furthermore, homeostatic macrophage (MΦ) clusters decreased while inflammatory MΦ and T-cell clusters increased with disease progression. A foamy MΦ cluster was increased in the fibrosa of mildly diseased tissues. Some side-dependent VEC clusters represented non-diseased, protective phenotypes, while others were CAVD-associated and were characterized by genes enriched in pathways of inflammation, endothelial–mesenchymal transition, apoptosis, proliferation, and fibrosis. Interestingly, we found several activator protein-1 (AP-1)-related transcription factors (*FOSB*, *FOS*, *JUN*, *JUNB*) and *EGR1* to be upregulated in the fibrosa and diseased aortic valve leaflets. Conclusions: Our results showed that VECs are highly heterogeneous in a side- and CAVD-dependent manner. Unique VEC clusters and their differentially regulated genes and pathways found in the fibrosa of diseased tissues may represent novel pathogenic mechanisms and potential therapeutic targets.

## 1. Introduction

Calcific aortic valve disease (CAVD) is a highly prevalent condition, particularly in the aging population (>65 years old), where its incidence exceeds 20% [1,2]. Risk factors include aging, obesity, male sex, diabetes, hyperlipidemia, smoking, and genetic predispositions, such as congenital defects. Despite recent advances in therapeutic target discovery, there is still a lack of medical therapy for early and progressing CAVD due to the gaps in the mechanistic understanding of its pathogenesis [3]. To date, CAVD is primarily treated in advanced stages by aortic valve repair or replacement using surgical, open-heart, or transcatheter methods [4]. Recent attempts to target some of the known risk factors, such as lipid-lowering therapies, have failed in clinical trials, highlighting a dire need for a better mechanistic understanding of the disease for drug development [5,6].

The highly complex biological landscape of the aortic valve includes several cellular and extracellular phenotypes and is actively regulated by mechanical forces in the onset and progression of CAVD [7,8,9]. Each aortic valve leaflet is composed of three distinct layers with unique extracellular matrix composition: the fibrosa, a collagen-rich layer facing the aorta, mostly composed of a monolayer of valvular endothelial cells (VECs) overlying valvular interstitial cells (VICs); the spongiosa, the middle layer, containing a high-glycosaminoglycan matrix with VICs; and the ventricularis, facing the left ventricle and enriched with elastin, composed of a VEC monolayer and VICs [10]. Emerging evidence suggests that each layer also includes resident macrophages (MΦ) [11]. CAVD encompasses a disease spectrum that begins with VEC dysfunction in non-diseased leaflets, leading to the accumulation of pro-inflammatory immune cells (MΦ and T cells), matrix remodeling, and leaflet thickening (aortic sclerosis). Furthermore, VECs’ differentiation into VIC phenotypes through endothelial–mesenchymal transition (EndMT) and VICs’ differentiation from quiescent states into activated, myofibroblastic, and osteogenic phenotypes lead to leaflet stiffening and aortic valve narrowing (aortic stenosis), producing impaired flow conditions and clinical events [12,13,14].

CAVD develops in a side-dependent manner, with VEC dysfunction, leaflet thickening, and fibrocalcification preferentially occurring in the fibrosa [15,16]. The underlying mechanisms for this side-dependent fibrocalcification pattern are unclear, but a leading hypothesis is that differential hemodynamic conditions, such as abnormal shear stress acting on VECs in the fibrosa as opposed to the high-magnitude, pulsatile pattern on the ventricularis VECs, are a major contributing factor [17,18]. Side-dependent differences in the various cell types and gene expression patterns in the aortic valve, particularly in VECs, may provide important insight into the cellular and molecular pathways that regulate CAVD and unveil novel therapeutic targets.

Prior studies delving into the underlying mechanisms and identification of potential therapeutic targets have used whole-leaflet tissue preparations for multi-OMICs studies, which have highlighted some important CAVD stage-associated markers. Recently, proteomic analyses of the aortic valve in healthy, fibrotic, and diseased stages revealed key layer-dependent changes [19]; however, the cells responsible for those changes could not be determined, due to the bulk protein lysis method used. Additionally, a recent single-cell RNA sequencing (scRNAseq) study comparing healthy and calcified human aortic valve leaflets identified various cell phenotype changes as CAVD progresses [20]. However, in that study, whole leaflets were digested to obtain single cells, resulting in a relatively small number of VECs analyzed (~4% of total cells; <1400 VECs in two clusters), making it impossible to determine the side-dependent VEC changes in CAVD at single-cell resolution. These results could not determine whether there are unique VEC populations in the fibrosa with pro-CAVD gene expression profiles.

In an scRNAseq study using the murine atherosclerosis model, we showed that disturbed flow, like that experienced by fibrosa VECs, induces endothelial reprogramming, involving endothelial inflammation, EndMT, and the novel endothelial–immune transition [21]. Here, we hypothesized that the preferential development of fibrocalcification in the fibrosa is mediated by specific VEC clusters with side-dependent gene expression patterns. To test this hypothesis, we isolated VEC-enriched single-cell preparations from the fibrosa side and the ventricularis side independently, and we carried out scRNAseq to identify side-dependent VEC clusters and genes at a single-cell resolution. Our study revealed 27 different cell clusters, including 7 VEC clusters, 9 VIC clusters, 1 stromal cell cluster, 3 transitional cell clusters, and 7 different immune cell clusters. Furthermore, we identified numerous side- and CAVD-dependent VEC sub-clusters, genes, and pathways. Overall, our scRNAseq study paints the aortic valve as a vastly heterogeneous and dynamic structure, with cells in constant communication, and provides further mechanistic insight for CAVD.

## 2. Methods

### 2.1. Side-Dependent, VEC-Enriched Single-Cell Isolation and Library Preparation for scRNAseq

Four sets of fresh human aortic valve leaflets were obtained from hearts not suitable for transplant, donated for research by the LifeLink Foundation. These leaflets were exempt from Institutional Review Board (IRB) approval as determined by the Emory University IRB. One set of leaflets was recovered from a patient undergoing aortic valve replacement surgery at Emory University Hospital and transported on ice to the lab for single-cell isolations. The collection and consent protocol was approved by the Emory University IRB under studies IRB00000149 and IRB00109646. Cell dissociation buffer was prepared as previously described [21]. Briefly, 500 units/mL of collagenase type I (MP Biomedical, Santa Ana, CA, USA), 500 units/mL of collagenase type II (MP Biomedical), 150 units/mL of collagenase type XI (Sigma-Aldrich, St. Louis, MO, USA), 60 units/mL of hyaluronase type Is (Sigma-Aldrich), and 60 units/mL of DNASE I (Zymo, Irvine, CA, USA) were dissolved in HBSS without calcium and magnesium, and then filtered through a 0.45 um syringe filter.

Our side-dependent single-cell isolation method was designed to test the hypothesis that calcific aortic valve disease occurs preferentially in the fibrosa due to the changes in gene expression patterns in specific VEC clusters in a side-dependent manner. To this end, we devised our cotton swabbing method to collect single cells enriched with VECs from the fibrosa side and the ventricularis side, independently. Briefly, one-and-a-half aortic valve leaflets from each patient were placed with the fibrosa face-up, and then dissociation buffer was placed on the upwards-facing layer and incubated for 45 min at 37 °C. The remaining one-and-a-half aortic valve leaflets from each patient were placed with the ventricularis side face-up and were also incubated with the dissociation buffer under the same conditions as the fibrosa side. After incubation, cells from the fibrosa and ventricularis sides (side-dependent VEC-enriched single-cell preparations) were gently scraped off using a sterile cotton swab, blocked with fetal bovine serum (FBS), and passed through a 70 μm cell strainer. The cell suspensions were centrifuged and resuspended in 2% BSA in PBS at a concentration of 1000 cells/μL. Following the side-dependent VEC-enriched single-cell preparations, the leftover tissues from each patient were pooled and further digested for an additional 30 min, treated with 2% FBS, and passed through a 70 μm cell strainer as well. Three different single-cell preparations (fibrosa, ventricularis, and leftover) from five patients were separately processed using the 10X Genomics chromium device (Pleasanton, CA, USA) for single-cell encapsulation. The cDNA libraries (total of 13 libraries: 3 libraries for each of the 5 patients, except for Donor #2’s ventricularis and Donor #5’s leftover) were prepared and sequenced on Illumina NovaSeq (San Diego, CA, USA) to a minimum depth of 25,000 reads per cell.

### 2.2. ScRNAseq, Differential Gene Expression, Gene Ontology, and Cell–Cell Interaction Analysis

The scRNAseq data files were processed and aligned to the human reference genome (GRCh38) using CellRanger from 10X Genomics. The generated h5 files for each sample were processed with the Seurat R package (v3) for further analysis [22]. Quality checks to retain cells with 200–8000 unique feature counts and less than 20% of mitochondrial counts were performed. All datasets were then merged, log-normalized, scaled, clustered, and visualized by uniform manifold approximation and projection for dimension reduction (UMAP). Raw and processed files and their respective metadata are accessible via NCBI GEO under accession number GSE220774.

Cell types were annotated using known markers for VECs, VICs, MΦ, and T-cells. Clusters with low expression of all annotation markers were designated as undefined stromal cells. Clusters with expression of more than 1 annotation marker subset were labeled as transitional cells. Multiple clusters of the same cell type were designated numbers (e.g., VEC1, VEC2).

Differentially expressed genes were identified as the top significantly (*p* < 0.05) up- or downregulated genes in each cluster relative to other clusters from the same cell type (e.g., VEC1 vs. VEC2–7). Gene Ontology analyses were performed using each cluster’s top 200 differentially expressed genes. The query list was uploaded to the Gene Ontology consortium website (http://geneontology.org), and the results were filtered to only include biological processes with *p*-values < 0.05 and a false discovery rate (FDR) < 0.05. Predictive cell–cell interaction analysis was performed using the CellChat package as per the developer’s instructions [23].

### 2.3. Aortic Valve Leaflet Fibrocalcification Assessment

Gross images of each aortic valve leaflet were taken from the fibrosa view (aortic side) and ventricularis view (ventricular side). The fibrosa view images were used to grade the fibrocalcification levels of each donor using a custom MATLAB (MathWorks, Natick, MA, USA) script to morphologically quantify the fibrosis (opaque yellow regions) and calcification (brown–white nodules) areas, normalized to the entire captured area of the leaflet surface.

### 2.4. Statistical Analysis

Data are shown as the mean ± SEM. The *n* numbers represent the number of cell plates or aortic valve leaflets. Non-parametric Mann–Whitney tests were performed. A *p*-value < 0.05 was considered statistically significant. All statistical analyses were performed using GraphPad Prism Version 9 (GraphPad, Boston, MA, USA).

## 3. Results

### 3.1. ScRNAseq Reveals That Cell Clustering Patterns in the Aortic Valve Vary with CAVD Stage

**Figure 1:**
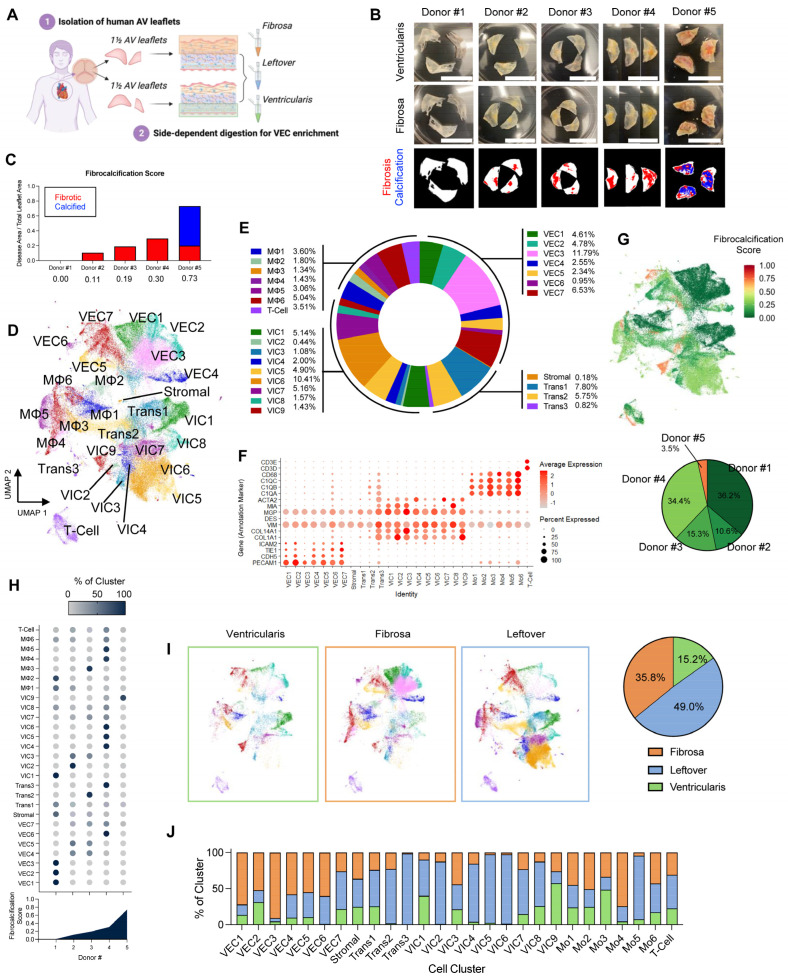
Side-dependent, VEC-enriched scRNAseq of human aortic valve reveals 27 cell clusters that vary with fibrocalcific stage: (**A**) Strategy for side-dependent, VEC-enriched scRNAseq of human aortic valve leaflets. (**B**) Gross images of aortic valve leaflets used for scRNAseq taken from ventricularis (top) and fibrosa (middle) views. The bottom row shows quantified segmentation images of the fibrotic (red) and calcified (blue) regions. Scale bar = 1 cm. (**C**) Quantification of fibrocalcification score for each donor. (**D**) UMAP of the 82,356 cells from the merged and filtered dataset, with labeled cell clusters. (**E**) Proportions of cell types (VECs, VICs, transitional, and immune) in the dataset. (**F**) Dot plot depicting average expression of annotation markers for each cell cluster. (**G**) Labeling of each cell by fibrocalcification score and proportion of cells per donor in the dataset. (**H**) Proportion of each cell cluster based on donor fibrocalcification score. (**I**) UMAP analysis of cell clusters from ventricularis, fibrosa, and leftover (mixed), and proportion of cells from each sample. (**J**) Side-dependent proportions in each cell cluster. VEC: valvular endothelial cell; scRNAseq: single-cell RNA sequencing; VIC: valvular interstitial cell; UMAP: uniform manifold approximation and projection.

Five fresh sets of human aortic valve leaflets were obtained from postmortem donors not suitable for transplantation or replacement surgery (Table 1). Aortic valve single-cell suspensions were prepared immediately following dissection or surgery using our digestion protocol to obtain VEC-enriched single-cell preparations independently from the fibrosa side and the ventricularis side of each donor (Figure 1A). The VEC-enriched single-cell preparations contained multiple cell types, including VECs and VICs. Following the initial digestion for VECs, the leftover tissues were pooled and further digested to prepare an additional mixed single-cell sample for each donor (leftover). Three separate single-cell libraries for the fibrosa, ventricularis, and leftover for each of five patients were prepared (a total of 13 cDNA libraries), except for one ventricularis from Donor #2 and one leftover from Donor #5 due to technical reasons. The 13 libraries were sequenced separately to a minimum depth of 25,000 reads per cell for each library. The aortic valve samples were ranked in order of disease progression, from least (Donor #1) to most diseased (Donor #5) (Figure 1B). To stratify the tissues by disease stage relative to each other, the extent of fibrosis and calcification of each leaflet set were determined using a fibrocalcification score as a semi-quantitative estimation of CAVD (Figure 1C). Donor #1, coincidentally the youngest donor, had non-diseased, nearly translucent leaflets (fibrocalcification score = 0) despite being hypertensive (Table 1). Donors #2 and #3 showed early signs of fibrosis, while Donor #4 showed moderate fibrosis and thickening. Donor #5 showed advanced calcific nodules, predominantly on the fibrosa (fibrocalcification score = 0.73). ScRNAseq data analysis was performed using Seurat v3 [22]. The raw cell numbers isolated per sample ranged from 574 to 16,478 (average = 5725 cells) and had mean reads per cell ranging from 24,861 to 463,765 (average = 127,473 mean reads per cell). All datasets were merged and quality controlled by filtering out the cells expressing gene counts of >8000 or <200 to exclude multiplets and empty droplets, respectively, and those with mitochondrial counts > 20% to remove damaged cells, resulting in a total of 82,356 cells used for downstream analysis (Table 1). Following log-normalization and scaling, the cells were grouped by unsupervised graph-based clustering and visualized using UMAP analysis (Figure 1D,E).

The UMAP analysis revealed 27 different cell clusters, including 7 VEC clusters, 9 VIC clusters, 1 stromal cell cluster, 3 transitional cell clusters, and 7 immune cell clusters. VECs, VICs, MΦ, and T cells were annotated using canonical marker genes (Figure 1F and Supplementary Figure S1A) [21,24,25,26,27]: VECs (*PECAM1*^+^, *CDH5*^+^, *TIE1*^+^, and *ICAM2*^+^), VICs (*COL1A1*^+^, *COL14A1*^+^, *VIM*^+^, and *DES*^−^), MΦ (*C1QA*^+^, *C1QB*^+^, *C1QC*^+^, *CD68*^+^), and T cells (*CD3D*^+^, *CD3E*^+^). Additional cell clusters that did not clearly fall into one of these canonical cell types were defined as transitional cells (Trans1–3) expressing markers of VECs, VICs, and MΦ, while the undefined stromal cell cluster showed low expression of all annotation markers. To further test similarities and differences across all cell clusters, correlation analyses were performed on the average gene expressions of the selected annotation markers (Supplementary Figure S1B). This analysis showed clear distinctions between each cell type, validating that the annotation markers used were appropriate.

To determine whether the cell clustering patterns correlated with relative disease stage, we first labeled each cell with its fibrocalcification score on the UMAP. This annotation showed a general correlation between cell clustering and their assigned fibrocalcification score, suggesting that the relative disease stratification affected the cell clustering patterns (Figure 1G). We further quantified the proportion of cell clusters based on donor fibrocalcification score to determine the correlation between each unsupervised cell cluster and disease stage, where increasing or decreasing proportion trends would indicate cell clusters that vary with disease progression. This analysis revealed 11 disease-stage-specific clusters, i.e., clusters in which >90% of cells map to a unique fibrocalcification score or a single donor (Figure 1H). To determine whether there was any significant donor-to-donor variability within individual clusters, we performed a correlation analysis of the average gene expression across donors for each of the 27 clusters (Supplementary Figure S2). This analysis showed that all VEC clusters originated from all donors, with varying proportions. Additionally, most VIC clusters were also found in all donors, except for VIC2 (not found in Donor #3), VIC8 (not found in Donor #5), and VIC9 (not found in Donor #1). In addition, this correlation analysis showed that cells within individual clusters are indistinguishable from each other, regardless of donor. These results confirm the absence of donor-dependent variability in cell clustering. Altogether, these results show that the disease stage affects cell clustering patterns in all samples.

### 3.2. Aortic Valve Cell Clustering Patterns Change in a Side-Dependent Manner

To determine whether there were any cell clustering pattern changes in a side-dependent manner, the UMAP was split into ventricularis-, fibrosa-, and leftover (mixed)-derived cells (Figure 1I). Visual comparison of the fibrosa and ventricularis revealed apparent side-dependent clustering pattern changes. For example, VEC3 and VEC6 were more abundant in the fibrosa, while VIC1 and VIC9 were more abundant in the ventricularis. To better define these side-dependent patterns, we first determined the number of cells originating from each side. Nearly half of the cells in our dataset (49%) originated from the leftover samples. Interestingly, ~35% of the cells came from the fibrosa samples, whereas ~15% came from the ventricularis samples. Furthermore, we determined the side-dependent proportions of each cell cluster and found that, on average, 59% of VECs originated from the fibrosa and 13% from the ventricularis. In contrast, 65% of VICs originated from the leftover sample, while 40% of immune cells originated from the fibrosa and 21% from the ventricularis (Figure 1J). These results are consistent with the idea that leaflet thickening occurs preferentially in the fibrosa. However, these results also indicate that side-dependent clustering patterns may be influenced by the different cell counts. Furthermore, to address whether there were any donor-specific differences in side-dependent cell clustering, we calculated the side-dependent proportions of cells from each donor (Supplementary Figure S3A). We found that different donors provided varying proportions of cells from the fibrosa, ventricularis, and leftover samples. These differences made it difficult to use a simple proportional analysis for deciphering the underlying reasons for the observed side-dependency, and further in-depth analysis into these clusters was required, as detailed below.

### 3.3. VEC Clusters Change in a CAVD-Stage-Dependent Manner

**Figure 2:**
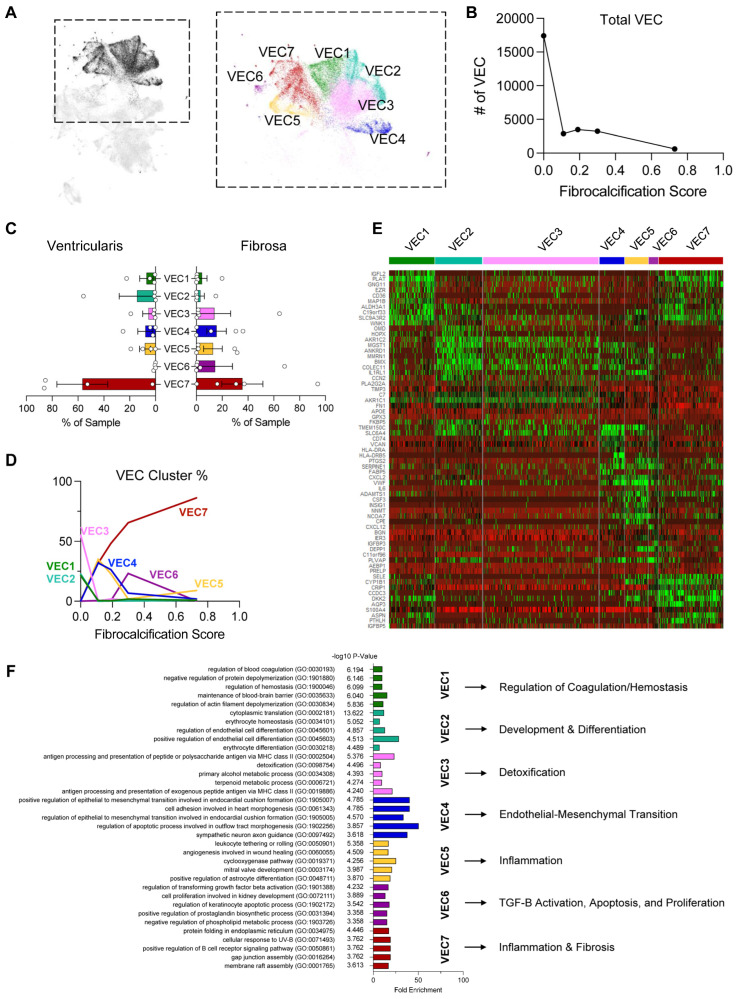
VEC clustering patterns change with disease stage: (**A**) UMAP of the 7 VEC clusters. (**B**) Total VEC numbers decrease with disease progression. (**C**) VEC cluster proportions in ventricularis vs. fibrosa. (**D**) VEC cluster proportions change as a function of disease stage. (**E**) Heatmap highlighting the top 10 most enriched genes in each VEC cluster. (**F**) Top 5 significantly enriched biological processes from a Gene Ontology analysis using the top 200 upregulated genes in each VEC cluster and a representative process for each cluster.

To determine the underlying factors for the changes in VEC clustering in a side- and disease-stage-dependent manner, we performed a VEC-specific analysis by sub-setting the seven VEC clusters found in our entire dataset (Figure 2A). Interestingly, we found that the total number of VECs decreased in a disease-stage-dependent manner, dramatically decreasing from non-diseased to fibrocalcific donors (Figure 2B). We also found no significant differences in the side-dependency of each VEC cluster (Figure 2C).

Interestingly, we found major differences in the VEC cluster proportions as the disease stage progressed (Figure 2D and Supplementary Figure S3B,C). The VEC1, VEC2, and VEC3 clusters were predominantly found in the non-diseased leaflets. Clusters VEC4 and VEC5 were found mostly in mildly fibrotic leaflets, whereas cluster VEC6 was found mostly in the moderately fibrotic leaflets. The VEC7 cluster progressively increased between the non-diseased and fibrocalcific leaflets, suggesting a clear and consistent disease-dependent trend for this cluster.

To determine the differentially expressed genes in each VEC cluster, we carried out a heatmap analysis using the 10 most enriched genes (Figure 2E). Interestingly, we found increased expression of *CD36*, which has been shown to be downregulated in aortic valve stenosis, in the VEC1 cluster [28]. VEC2 and VEC3 had the highest expression of *OMD*, known to slow down calcification [29], while VEC4 had the highest expression of *CD74*, which increases in static AVs relative to AVs exposed to physiological mechanical forces [30]. VEC4, along with VEC5, also had high expression of known pro-hemostatic and pro-calcific *VWF* [31]. VEC5 also had the highest expression of *IL6*, which has been identified by genome-wide association studies as being upregulated in aortic valve disease [32]. Meanwhile, VEC7 had the highest expression of *SELE*, which has been previously shown by scRNAseq to be involved in inflammation and potentially EndMT in a subset of VECs [20]. To further characterize each cluster, we performed a Gene Ontology analysis using the top 200 upregulated genes in each cluster and highlighted the most representative and potentially relevant biological and pathobiological processes in CAVD (Figure 2F). This analysis suggested that the enriched pathways are regulation of coagulation/hemostasis in VEC1, developmental and differentiation processes in VEC2, detoxification in VEC3, EndMT in VEC4, inflammation in VEC5, apoptosis and proliferation in VEC6, and fibrosis and inflammation in VEC7. Together, these results suggest that clusters VEC1–3 are associated with protective phenotypes, while VEC4–7 are associated with pathogenic phenotypes.

### 3.4. Identification of Side- and CAVD-Stage-Dependent Genes in VECs

**Figure 3:**
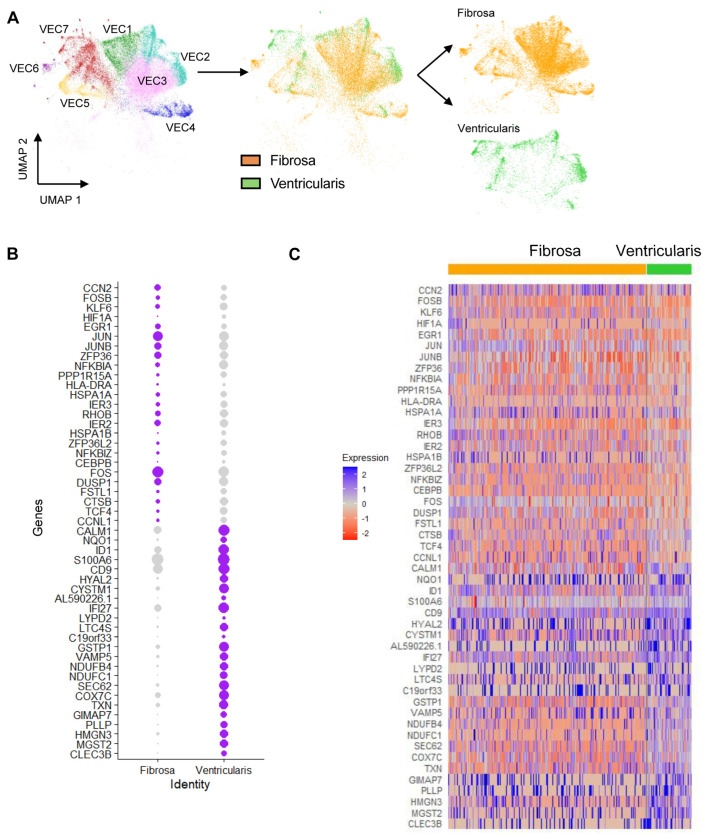
Identification of side-dependent VEC clustering patterns and differentially expressed genes (DEGs): (**A**) Side-dependent separation of VEC UMAP into fibrosa vs. ventricularis. (**B**) Side-dependent expression of DEGs in fibrosa or ventricularis VECs. (**C**) Heatmap classifying side-dependent DEGs in fibrosa or ventricularis VECs. Genes upregulated in the fibrosa of diseased tissues and genes upregulated in the ventricularis of non-calcified tissues are indicated.

To further determine whether VECs change in a side-dependent manner, we first selected VECs from the fibrosa-enriched and ventricularis-enriched samples and separated the UMAP by its side of origin (Figure 3A). This UMAP showed clear separation of VECs from the fibrosa and ventricularis within individual clusters, suggesting side-dependent transcriptional differences. To unveil the subset of side-dependent genes, we performed a differential gene expression analysis between the fibrosa and ventricularis VECs. In total, we identified 49 top side-dependent genes (Figure 3B,C). We then compared this list of genes to the CAVD stage using the fibrocalcification score. Interestingly, in the top subset of genes upregulated in the fibrosa in diseased leaflets, we found several members of the activator protein-1 (AP-1) transcription factor (*FOSB*, *EGR1*, *JUN*, and *JUNB*), indicating its potential correlation with CAVD.

**Figure 4:**
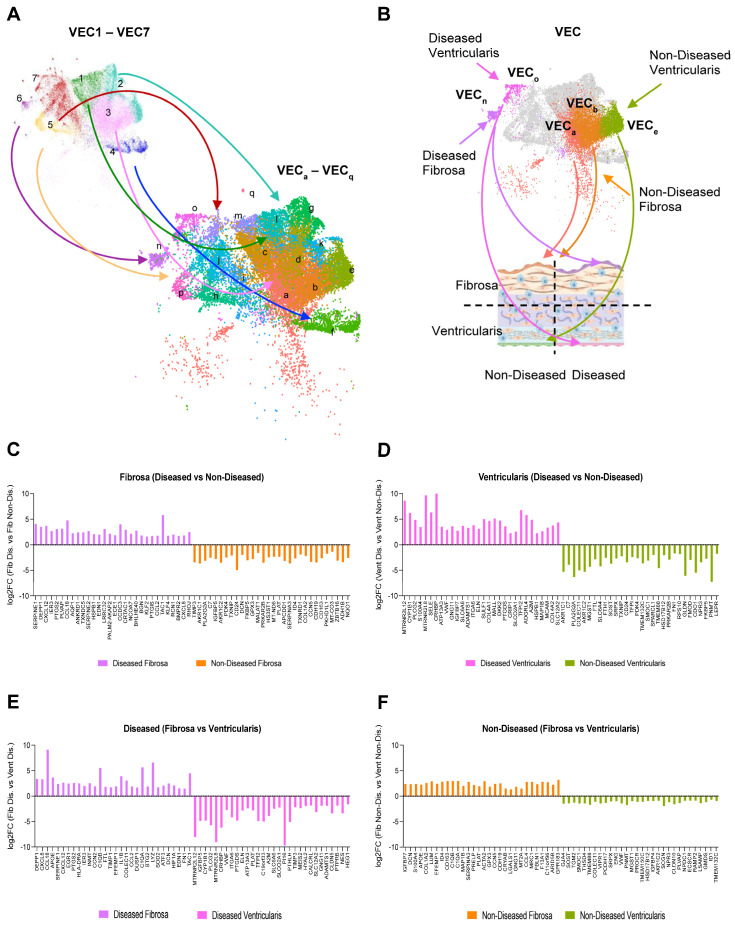
Identification of side- and disease-dependent sub-clusters of VECs and genes: (**A**) Seven VEC clusters (VEC1–7) were divided into 17 sub-clusters (VECa–q). (**B**) Identification of diseased or non-diseased, side-dependent sub-clusters of VECs (non-diseased fibrosa = sub-clusters a and b, non-diseased ventricularis = sub-cluster e, diseased fibrosa = sub-cluster n, and diseased ventricularis = sub-cluster o). (**C**,**D**) Top 30 DEGs between diseased and non-diseased VEC in the fibrosa (**C**) and ventricularis (**D**) sub-clusters. (**E**,**F**) Top 30 DEGs between fibrosa and ventricularis VECs in diseased (**E**) and non-diseased (**F**) sub-clusters.

Given the clear separation between some of the fibrosa and ventricularis VEC clusters, we performed a sub-clustering analysis of the original seven VEC subsets to further validate side- and disease-dependent expression of VEC genes (Figure 4A). The original seven VEC clusters were divided into 17 sub-clusters, 12 of which we determined to be side-dependent (Supplementary Figure S4A–C). Then, these side-dependent VEC sub-clusters were cross-checked for disease-dependency using the original seven VEC cluster designations (Supplementary Figure S4D), and for their CAVD stage using the fibrocalcification score (Supplementary Figure S4E). Using this information, we determined a combination of sub-clusters that represent non-diseased or diseased VECs from either the fibrosa or ventricularis: VEC_a_ and VEC_b_ = non-diseased fibrosa, VEC_e_ = non-diseased ventricularis, VEC_n_ = diseased fibrosa, and VEC_o_ = diseased ventricularis (Figure 4B). Using these sub-clusters, we identified differentially expressed genes in a disease- (Figure 4C,D) and side-dependent manner (Figure 4E,F). This analysis revealed several chemokines and cytokines, including *CXCL8*, *CCL18*, *CXCL12*, *CCL2*, and *IL1B*, as the most upregulated genes in the diseased fibrosa VEC sub-cluster, as well as genes with potential roles in EndMT, such as *CCN2* and *HIF1A*, endothelial–immune transition, such as *C1QA*, *C1QB*, and *LYZ*, and hemostasis, such as *SERPINE1* and *EGR1* [33,34]. Interestingly, the AP-1 complex members identified in Figure 4 were also significantly upregulated in the diseased fibrosa VEC sub-cluster; however, they were not classified among the top 30 genes reported here. A Gene Ontology analysis using the top 200 differentially expressed genes in each VEC sub-cluster revealed increased pro-migratory, pro-angiogenic, and pro-inflammatory processes in the diseased and fibrosa-derived VECs, whereas biosynthetic processes were enriched in the non-diseased ventricularis VECs (Supplementary Figure S5). Pseudotime analysis indicated gene expression pattern transitions from the non-diseased ventricularis VECs to non-diseased fibrosa VECs, ending in diseased VECs from both sides of the aortic valve (Supplementary Figure S6). Together, these analyses revealed side- and disease-stage-dependent VEC genes that represent potential therapeutic targets in CAVD.

### 3.5. ScRNAseq Shows That VICs and Immune Cells Are Regulated in a CAVD-Stage-Dependent Manner

**Figure 5:**
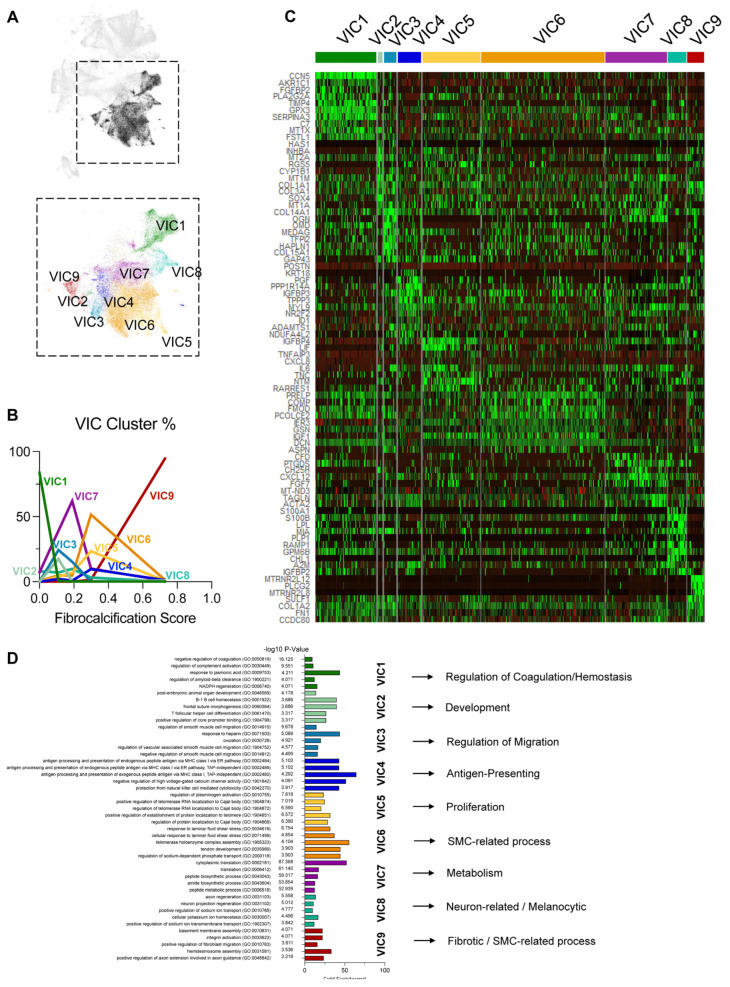
VIC clustering patterns change with disease stage: (**A**) UMAP of the 9 VIC clusters. (**B**) VIC cluster proportions change as a function of disease stage. (**C**) Heatmap highlighting the top 10 most enriched genes in each VIC cluster. (**D**) Top 5 significantly enriched biological processes from a Gene Ontology analysis using the top 200 upregulated genes in each VIC cluster and a representative process for each cluster.

In addition to VECs, our scRNAseq study also revealed other aortic valve cell types: nine VIC clusters, six MΦ clusters, one T-cell cluster, three transitional cell clusters, and one stromal cell cluster (Figure 1). We first determined whether the VIC clusters differed in a CAVD-dependent manner. Non-diseased leaflet VICs were primarily composed of VIC1, mildly fibrotic leaflets had the highest proportions of VIC2, VIC3, and VIC7, and the moderately fibrotic leaflets had the largest fraction of VIC5 and VIC6 (Figure 5A,B). Most of the fibrocalcified leaflet VICs belonged to the VIC9 cluster, while the relative proportions of VIC4 and VIC8 remained similar throughout most tissues. This demonstrated that VIC clusters change in a CAVD-dependent manner.

The heatmap analysis showed unique gene expression patterns of each VIC cluster (Figure 5C). We found that VIC1 had the highest expression of *CCN5*, a known anti-calcific gene [35]. VIC4 had the highest expression of smooth muscle cell markers, indicative of VIC activation. VIC5–7 had the highest expression of pro-inflammatory *IL6*, while VIC5 and VIC6 also had the highest expression of *COMP*, known to be involved in cartilage development [36]. One of the most differentially expressed genes in VIC9 was *PLCG2*, which has been implicated in osteoclastogenesis and protection against Alzheimer’s disease [37,38,39].

A Gene Ontology analysis suggested that the notable enriched pathways were anti-hemostatic in VIC1, homeostatic regulation and development in VIC2, migration in VIC3, antigen-presenting processes in VIC4, proliferation in VIC5, SMC-related processes in VIC6, metabolic processes in VIC7, neuronal processes (possibly retained from development) in VIC8, and barrier function and fibrosis in VIC9 (Figure 5D).

Immune cell proportions varied greatly across CAVD stages (Supplementary Figure S7A,B). Strikingly, the overall proportions of MΦ1 and MΦ2 decreased from the non-diseased to fibrocalcific leaflets, while mildly fibrotic leaflets were primarily composed of MΦ3. The moderately fibrotic and fibrocalcific leaflets had the highest proportions of MΦ5, and the fibrocalcific leaflets alone had the highest fraction of T cells. The relative proportions of MΦ4 and MΦ6 did not display a clear disease-dependent trend. Further characterization of immune cell clusters was performed through differential gene expression analysis (Supplementary Figure S7C) and using canonical markers of M1/M2 MΦ, resident-like MΦ, and subtypes of inflammatory MΦ [40,41]. These analyses did not reveal a clear M1 or M2 MΦ cluster (Supplementary Figure S7D). However, we found that MΦ3, MΦ4, and MΦ5 represented resident-like, foamy, and inflammatory MΦ, respectively (Supplementary Figure S7E). A Gene Ontology analysis (Supplementary Figure S7F) further revealed that the enriched pathways were metabolic in MΦ1, mitotic in MΦ2, and inflammatory and foamy in MΦ6. Transitional cells and stromal cells, representing a fraction of cells with unclear gene signatures and Gene Ontology, showed no clear or CAVD-dependent patterns (Supplementary Figure S8).

There were no apparent side-dependent VIC clusters (Supplementary Figure S9A,B). Only one MΦ cluster, foamy MΦ4, indicated a fibrosa-specific accumulation (Supplementary Figure S9C,D). Its importance is unclear and warrants further study; however, its side-dependent presence correlates with the preferential fibrosa-specific pattern of inflammation and subsequent calcification. Transitional cell clusters also did not reflect any clear side-dependent patterns (Supplementary Figure S9E,F).

### 3.6. Cell–Cell Interaction Analysis to Predict Mechanisms in CAVD

We used the predictive cell–cell interaction package CellChat to infer potential communication patterns, with emphasis on VEC–VIC, VEC–immune, and VIC–immune signaling [23]. We identified 123 potential signaling pathways across all clusters (Supplementary Figure S10A), which were further grouped into three outgoing and three incoming communication patterns, primarily composed of VECs, VICs, or immune cells (Supplementary Figure S10B,C). To narrow down a list of relevant signaling pathways, we extracted the list of statistically significant interactions with outgoing VEC and incoming VIC signaling (Supplementary Figure S10D), outgoing VEC and incoming immune signaling (Supplementary Figure S10E), and outgoing VIC and incoming immune signaling (Supplementary Figure S10F). VEC-VIC interactions through signaling pathways known to increase during valvular development, such as WNT and NRG, were upregulated in inflammatory and fibrotic VECs, suggesting additional roles in disease progression [7,42,43]. Interestingly, developmental VEC2 and inflammatory VEC5 and VEC7 were predicted to interact with the receptor DAG1 across various VIC clusters through either HSPG2 or AGRN, respectively. This could be representative of a switch between protective (VEC2-VIC) and inflammatory (VEC5/7-VIC) signaling. Additionally, PTHLH signaling, known to regulate bone formation and resorption [44], was predicted to be upregulated between VEC1, VEC5, VEC7, VIC1, and VIC8. Furthermore, pro-inflammatory VEC5 were predicted to interact with foamy MΦ6 via CSF3-CSF3R. These findings suggest potential cell–cell interactions among various aortic valve cell types relevant in CAVD’s pathogenesis. RNA velocity analysis did not indicate any clear side- and disease-dependent transitions from one VEC or VIC sub-cluster to others, but it did indicate potential transitions from homeostatic to pro-inflammatory MΦ as well as transitions away from trans cells (Supplementary Figure S11).

### 3.7. Identification of AP-1-Related Transcription Factors as Side-Dependent Genes in VECs and Potential Therapeutic Targets

**Figure 6:**
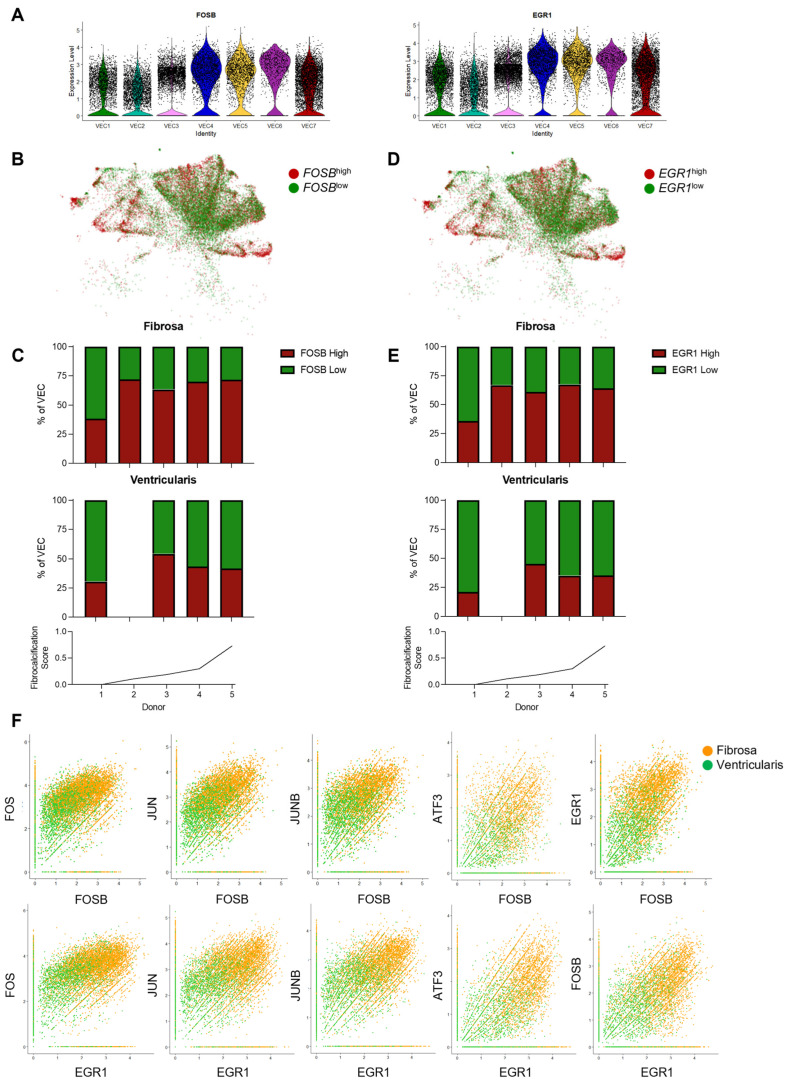
*FOSB* and *EGR1* are upregulated in fibrosa VECs in diseased aortic valve leaflets: (**A**) Violin plots showing that *FOSB* and *EGR1* expression is increased in disease-associated VEC clusters. (**B**) VEC UMAP separated by high *FOSB* expression or low *FOSB* expression (average relative *FOSB* expression = 1.5). (**C**) Disease-stage-associated proportions of *FOSB*^high^ and *FOSB*^low^ VECs, split in a side-dependent manner. (**D**) UMAP of the VECs separated by high *EGR1* expression or low *EGR1* expression (average relative *EGR1* expression = 1.5). (**E**) Stacked bar graph of individual donor proportions of *EGR1*^high^ and *EGR1*^low^ VECs, split by layer of origin. (**F**) VECs from the fibrosa (orange) and ventricularis (green) show co-expression of *FOSB* (**top row**) or *EGR1* (**bottom row**) with other AP-1-related transcription factors (*FOS*, *JUN*, *JUNB*, *ATF3*, *FOSB*, and *EGR1*).

Our results so far have identified several VEC genes that are differentially upregulated in a side- and disease-stage-dependent manner. Most interestingly, several of these genes, including the AP-1 complex-related transcription factors *FOSB* and *EGR1*, exhibited the expected reduced expression patterns in the protective VEC1–VEC3 and upregulation in the more CAVD-associated VEC4–VEC7 (Figure 6A and Supplementary Figure S12A). Additional analysis showed that the *FOSB*^high^ and *EGR1*^high^ VEC proportions in the fibrosa were increased in the fibrotic and fibrocalcified leaflets compared to the non-diseased leaflets (Figure 6B–E). Meanwhile, the proportions of *FOSB*^high^ and *EGR1*^high^ VECs in the ventricularis remained relatively constant across the disease stratifications. Furthermore, co-expression analyses showed that VECs from the fibrosa highly co-expressed *FOSB*, *EGR1*, and other AP-1-complex-related transcription factors such as *FOS*, *JUN*, *JUNB*, and *ATF3* (Figure 6F), whereas VECs from the ventricularis did not, suggesting their regulatory potential in the side-dependent pathophysiology of CAVD.

## 4. Discussion

Here, we tested whether there were VEC populations unique to the fibrosa vs. ventricularis in a CAVD-stage-dependent manner by scRNAseq. We analyzed 82,356 cells obtained from the fibrosa vs. ventricularis sides using fresh human aortic valve leaflets ranging in age and CAVD severity. Our findings revealed 27 distinct cell clusters, including 7 VEC clusters, 9 VIC clusters, 1 stromal cell cluster, 3 transitional cell clusters, and 7 immune cell clusters, which changed in a side- and disease-dependent manner. We also found that clusters VEC1–3 represent protective phenotypes, while clusters VEC4–7 are CAVD-associated and are characterized by genes enriched in pathways of inflammation, EndMT, apoptosis, proliferation, and fibrosis. Furthermore, we identified many differentially expressed genes in VECs, in a side- and CAVD-dependent manner, including several AP-1-complex-related transcription factors upregulated in the fibrosa and CAVD.

To determine whether there were any side-dependent VEC populations in a CAVD-stage-dependent manner, we first established a new protocol that enabled us to obtain single-cell suspensions enriched with VECs specifically from either the fibrosa side or the ventricularis side of fresh human aortic valve leaflets. Using this protocol, we were able to collect 82,356 cells for analysis, of which 27,627 were VECs (~34% of total cells). This large number of VEC allowed us to identify seven VEC clusters (VEC1–7) expressing canonical endothelial markers (*PECAM1*^+^, *CDH5*^+^, *TIE1*^+^, *ICAM2*^+^), which indicated apparent side- and disease-stage-dependent differences. Clusters VEC1–3, which were mostly found in non-diseased tissues, were found to be homeostatic and protective. On the other hand, clusters VEC4–7, found mostly in diseased tissues, were enriched with pathways related to CAVD’s pathogenesis, including endothelial–mesenchymal transition, inflammation, TGF-B activation, apoptosis, proliferation, and fibrosis (Figure 2F). To further define side- and disease-stage-dependent differences, the VEC clusters were classified into 17 sub-clusters (VEC_a–q_) to perform detailed downstream gene and pathway analyses.

Consistent with our initial hypothesis, our results showed several side- and disease-dependent sub-clusters (Figure 4A). Of the 17 total VEC sub-clusters, VEC_e_ was specific to non-diseased ventricularis, VEC_o_ to diseased ventricularis, VEC_a_ and VEC_b_ to non-diseased fibrosa, and VEC_n_ to diseased fibrosa. Additionally, five VEC sub-clusters (VEC_f_, VEC_h_, VEC_i_, VEC_k_, and VEC_l_) were common across both sides, while seven sub-clusters (VEC_c_, VEC_d_, VEC_g_, VEC_j_, VEC_m_, VEC_p_, and VEC_q_) showed side-dependency but not disease-stage-dependency.

We also noted that VEC_e_ vs. VEC_a,b_ sub-clusters were found in a side-dependent manner in non-diseased conditions. This was likely due to microenvironmental differences, such as shear stress, to which these VECs were exposed to for decades [17,18]. While these VECs were found in non-diseased tissues, the fibrosa-specific VECs (VEC_a,b_) showed upregulation of markers of immune cells (*C1QA*, *C1QB*, *CD163*), the cytokine *CCL4*, and the mesenchymal markers *LUM* and *DCN* (Figure 4E), suggesting the presence of endothelial inflammation and early stages of EndMT in the fibrosa of an environment lacking fibrocalcification. Furthermore, compared to non-diseased conditions, the VEC sub-clusters in diseased tissues (VEC_n_ and VEC_o_) showed even more side-dependent upregulation of *LYZ*, inflammatory cytokines and chemokines (*CXCL8*, *CCL18*, *CXCL12*, *IL1B*, and *CCL2*), and EndMT markers (*HIF1A* and *CCN2*) in the fibrosa (Figure 4D). These gene expression patterns were similar to those observed in endothelial reprogramming, characterized by endothelial inflammation, EndMT, and the novel endothelial–immune transition, in response to disturbed flow occurring in the arteries of mice [21]. Whether VECs in the fibrosa undergo endothelial reprogramming needs further validation, but if true, this may be a crucial mechanism for side-dependent CAVD pathophysiology, and these genes could represent potential therapeutic targets.

Our phenotypic assignment of each cell cluster was an in silico prediction based on the analysis of the scRNAseq data. Its importance lies in its generation of numerous hypotheses that, in turn, need to be validated by additional approaches. Whether these predicted phenotypic changes—such as inflammatory, apoptotic, and proliferative responses of aortic valve cells—occur in CAVD should be validated in the future. Moreover, it also needs to be studied whether they play important roles in CAVD’s development.

Our study revealed AP-1 complex members (*FOSB*, *FOS*, *JUN*, *JUNB*) and *EGR1* as side- and disease-dependent genes. *EGR1* was shown to be upregulated in aortic valve stenosis [33], but AP-1 complex members have not been reported in the context of CAVD. Testing the role of AP-1-specific inhibitors would be interesting to determine whether they could prevent or reverse CAVD.

While we focused on testing our main hypothesis that VEC clusters and phenotypes change in a side- and disease-state-dependent manner, we found that VICs and immune cells also change in a disease-state-dependent manner. As the disease progressed, VIC clusters enriched with homeostatic pathways (VIC1–3 and 7) were reduced, while VIC clusters enriched with fibrocalcific pathways (VIC9) increased (Figure 5). Additionally, the homeostatic MΦ1 and MΦ2 clusters decreased while the inflammatory MΦ5 and T cells increased with disease progression (Supplementary Figure S7). Increases in VIC9, MΦ5, and T cells, in diseased tissues indicate their potential role in CAVD. The genes that were highly increased in VIC9, such as *PLCG2*, *MTRNR2L12*, and *MTRNR2L8*, represent potential therapeutic targets (Figure 5C) [37,45].

## 5. Potential Limitations

The results reported here have several limitations and should be interpreted with some caution. We used a small number of donors from males only, and Donor #5 was the only sample with extensive calcification. The remaining tissues from Donors #2, 3, and 4, but not Donor #1, showed varying degrees of fibrosis (Figure 1B,C). This limitation should be overcome with additional patient samples in the future. We also noted that twice as many total single cells were obtained from the fibrosa (~35%) compared to the ventricularis (~15%). Moreover, we found that ~59% of VECs originated from the fibrosa and ~13% from the ventricularis. It is unclear whether this reflects an inherent difference in total cell numbers, including VECs, between the fibrosa and the ventricularis, or side-dependent susceptibility to the digestion conditions. We also observed that the total VEC count decreased as CAVD progressed, based on the fibrocalcification score. It is unknown whether this reduction in the number of VECs indeed occurs as the disease progresses, or whether it is caused by a change in digestion susceptibility due to the varying extent of fibrocalcification. While the underlying causes for these cell count differences require further study, we took these differences into careful consideration in our downstream analyses (Supplementary Figures S3 and S4). To address these concerns, specific marker findings should be validated by immunostaining new, independent aortic valve samples from different donors than those used in the scRNAseq study. In addition, we also conducted spatial transcriptomics studies multiple times using the 10X Genomics Visium platform. Unfortunately, we were unable to obtain satisfactory results due to the poor RNA quality of the freshly obtained aortic valves from multiple patients. We are continuing to optimize this methodology to overcome the technical issues to generate good-quality samples of aortic valve tissues (across the disease spectrum) for spatial transcriptomics. These technologies will help to further define the side- and disease-dependent gene expression patterns and their therapeutic potential.

In summary, our scRNAseq study revealed that VECs are highly heterogeneous, showing side- and disease-dependent gene expression patterns in human aortic valve leaflets with varying degrees of CAVD. We identified unique VEC clusters in the fibrosa of diseased tissues. The highly abundant genes and pathways in these VECs, such as AP-1 genes, represent potential novel pathogenic mechanisms and therapeutic targets.

## Figures and Tables

**Table 1:** Donor demographics and scRNAseq sample information.

Donor Number	Age	Race	Sex	COD	Hyper-Tension	Social Hx	Clinical Hx	FC Score	Sample	Number of Cells	Mean Reads per Cell
1	36	Black	M	CVA/Stroke	Y	Alcohol (Liq. 3 pt/d/2 y)	EF 35–40%	0.0	Fibrosa	16,748	27,548
									Leftover	7162	55,500
									Ventricularis	6571	68,801
2	48	Black	M	Anoxia	Y	Alcohol (1–2/3 m)	EF 25% MI, CHF	0.11	Fibrosa	3970	143,198
									Leftover	5697	127,581
									Ventricularis	N/A	N/A
3	54	White	M	CVA/Stroke	Y	Smoking (1 p/d/40 y) Alcohol (Beer 6 p/2 w/5 y) Drug Use (Various)	EF 65%	0.19	Fibrosa	4753	101,785
									Leftover	5609	88,879
									Ventricularis	3269	141,170
4	58	Hispanic	M	CVA/Stroke	Y	Smoking (1/2 cig/d/2 y) Alcohol (Beer 1–2/w/10 y)	Diabetes (3 y)	0.30	Fibrosa	4307	68,672
									Leftover	23,371	26,344
									Ventricularis	1209	442,804
5	60	White	M	N/A	Y	Smoking (unk)	Diabetes (unk) EF 60% Aortic Valve Disease	0.73	Fibrosa	574	463,765
									Leftover	N/A	N/A
									Ventricularis	2640	103,365

ScRNAseq: single-cell RNA sequencing; COD: cause of death; Hx: history, FC: fibrocalcification; CVA: cerebrovascular accident; EF: ejection fraction; MI: myocardial infarction; CHF: congestive heart failure; N/A: Not Applicable.

## Data Availability

The datasets presented in this study can be found in online repositories (GEO Accession GSE220774).

## Supplementary Materials

The following supporting information can be downloaded at https://www.mdpi.com/article/10.3390/genes15121623/s1: Figure S1: Annotation markers for cell clustering; Figure S2: No significant batch effect of single-cell RNA sequencing found between donors; Figure S3: VEC clustering patterns in each donor; Figure S4: Sub-clustering analysis of VEC; Figure S5: Gene ontology analysis of side- and disease stage-dependent sub-clusters; Figure S6: Diffusion map and pseudotime analysis of side- and disease stage-dependent VEC sub-clusters; Figure S7: Immune cell clustering patterns change with disease stage; Figure S8: Transitional cell clustering patterns change with disease stage; Figure S9: Side-dependent clustering analysis of VIC, immune, and transitional cells; Figure S10: Predictive cell-cell interaction analyses with CellChat; Figure S11: RNA Velocity analysis to predict cell cluster transitions; Figure S12: Side-dependent genes upregulated in the VEC fibrosa are predominantly increased in disease-associated VEC clusters (VEC4–7).

## References

[1] Virani S.S., Alonso A., Aparicio H.J., Benjamin E.J., Bittencourt M.S., Callaway C.W., Carson A.P., Chamberlain A.M., Cheng S., Delling F.N. (2021). Heart disease and stroke statistics-2021 update: A report from the american heart association. Circulation.

[2] Yadgir S., Johnson C.O., Aboyans V., Adebayo O.M., Adedoyin R.A., Afarideh M., Alahdab F., Alashi A., Alipour V., Arabloo J. (2020). Global, regional, and national burden of calcific aortic valve and degenerative mitral valve diseases, 1990–2017. Circulation.

[3] Kraler S., Blaser M.C., Aikawa E., Camici G.G., Luscher T.F. (2022). Calcific aortic valve disease: From molecular and cellular mechanisms to medical therapy. Eur. Heart J..

[4] Otto C.M., Nishimura R.A., Bonow R.O., Carabello B.A., Erwin J.P., Gentile F., Jneid H., Krieger E.V., Mack M., McLeod C. (2021). 2020 ACC/AHA guideline for the management of patients with valvular heart disease: A report of the American College of Cardiology/American Heart Association Joint Committee on Clinical Practice Guidelines. Circulation.

[5] Nsaibia M.J., Devendran A., Goubaa E., Bouitbir J., Capoulade R., Bouchareb R. (2022). Implication of lipids in calcified aortic valve pathogenesis: Why did statins fail?. J. Clin. Med..

[6] Pawade T.A., Doris M.K., Bing R., White A.C., Forsyth L., Evans E., Graham C., Williams M.C., van Beek E.J.R., Fletcher A. (2021). Effect of denosumab or alendronic acid on the progression of aortic stenosis: A double-blind randomized controlled trial. Circulation.

[7] Dutta P., Lincoln J. (2018). Calcific aortic valve disease: A developmental biology perspective. Curr. Cardiol. Rep..

[8] Villa-Roel N., Ryu K., Jo H., Aikawa E., Hutcheson J.D. (2020). Role of biomechanical stress and mechanosensitive miRNAs in calcific aortic valve disease. Cardiovascular Calcification and Bone Mineralization.

[9] Arjunon S., Rathan S., Jo H., Yoganathan A.P. (2013). Aortic valve: Mechanical environment and mechanobiology. Ann. Biomed. Eng..

[10] Yip C.Y., Simmons C.A. (2011). The aortic valve microenvironment and its role in calcific aortic valve disease. Cardiovasc. Pathol..

[11] Kim A.J., Xu N., Yutzey K.E. (2021). Macrophage lineages in heart valve development and disease. Cardiovasc. Res..

[12] Ma X., Zhao D., Yuan P., Li J., Yun Y., Cui Y., Zhang T., Ma J., Sun L., Ma H. (2020). Endothelial-to-mesenchymal transition in calcific aortic valve disease. Acta Cardiol. Sin..

[13] Yutzey K.E., Demer L.L., Body S.C., Huggins G.S., Towler D.A., Giachelli C.M., Hofmann-Bowman M.A., Mortlock D.P., Rogers M.B., Sadeghi M.M. (2014). Calcific aortic valve disease: A consensus summary from the Alliance of Investigators on Calcific Aortic Valve Disease. Arterioscler. Thromb. Vasc. Biol..

[14] Horne T.E., VandeKopple M., Sauls K., Koenig S.N., Anstine L.J., Garg V., Norris R.A., Lincoln J. (2015). Dynamic heterogeneity of the heart valve interstitial cell population in mitral valve health and disease. J. Cardiovasc. Dev. Dis..

[15] Holliday C.J., Ankeny R.F., Jo H., Nerem R.M. (2011). Discovery of shear- and side-specific mRNAs and miRNAs in human aortic valvular endothelial cells. Am. J. Physiol. Heart Circ. Physiol..

[16] Ankeny R.F., Thourani V.H., Weiss D., Vega J.D., Taylor W.R., Nerem R.M., Jo H. (2011). Preferential activation of SMAD1/5/8 on the fibrosa endothelium in calcified human aortic valves-association with low BMP antagonists and SMAD6. PLoS ONE.

[17] Driscoll K., Cruz A.D., Butcher J.T. (2021). Inflammatory and biomechanical drivers of endothelial-interstitial interactions in calcific aortic valve disease. Circ. Res..

[18] Fernandez Esmerats J., Villa-Roel N., Kumar S., Gu L., Salim M.T., Ohh M., Taylor W.R., Nerem R.M., Yoganathan A.P., Jo H. (2019). Disturbed flow increases UBE2C (ubiquitin E2 ligase C) via loss of miR-483-3p, inducing aortic valve calcification by the pVHL (von Hippel-Lindau protein) and HIF-1alpha (hypoxia-inducible factor-1alpha) pathway in endothelial cells. Arterioscler. Thromb. Vasc. Biol..

[19] Schlotter F., Halu A., Goto S., Blaser M.C., Body S.C., Lee L.H., Higashi H., DeLaughter D.M., Hutcheson J.D., Vyas P. (2018). Spatiotemporal multi-omics mapping generates a molecular atlas of the aortic valve and reveals networks driving disease. Circulation.

[20] Xu K., Xie S., Huang Y., Zhou T., Liu M., Zhu P., Wang C., Shi J., Li F., Sellke F.W. (2020). Cell-type transcriptome atlas of human aortic valves reveal cell heterogeneity and endothelial to mesenchymal transition involved in calcific aortic valve disease. Arterioscler. Thromb. Vasc. Biol..

[21] Andueza A., Kumar S., Kim J., Kang D.W., Mumme H.L., Perez J.I., Villa-Roel N., Jo H. (2020). Endothelial reprogramming by disturbed flow revealed by single-cell RNA and chromatin accessibility study. Cell Rep..

[22] Stuart T., Butler A., Hoffman P., Hafemeister C., Papalexi E., Mauck W.M., Hao Y., Stoeckius M., Smibert P., Satija R. (2019). Comprehensive integration of single-cell data. Cell.

[23] Jin S., Guerrero-Juarez C.F., Zhang L., Chang I., Ramos R., Kuan C.-H., Myung P., Plikus M.V., Nie Q. (2021). Inference and analysis of cell-cell communication using CellChat. Nat. Commun..

[24] Rutkovskiy A., Malashicheva A., Sullivan G., Bogdanova M., Kostareva A., Stenslokken K.O., Fiane A., Vaage J. (2017). Valve interstitial cells: The key to understanding the pathophysiology of heart valve calcification. J. Am. Heart Assoc..

[25] Chiyoya M., Seya K., Yu Z., Daitoku K., Motomura S., Imaizumi T., Fukuda I., Furukawa K.I. (2018). Matrix Gla protein negatively regulates calcification of human aortic valve interstitial cells isolated from calcified aortic valves. J. Pharmacol. Sci..

[26] Hutcheson J.D., Schlotter F., Creager M.D., Li X., Pham T., Vyas P., Higashi H., Body S.C., Aikawa M., Singh S.A. (2021). Elastogenesis correlates with pigment production in murine aortic valve leaflets. Front. Cardiovasc. Med..

[27] Latif N., Sarathchandra P., Chester A.H., Yacoub M.H. (2015). Expression of smooth muscle cell markers and co-activators in calcified aortic valves. Eur. Heart J..

[28] Syväranta S., Alanne-Kinnunen M., Oörni K., Oksjoki R., Kupari M., Kovanen P.T., Helske-Suihko S. (2014). Potential pathological roles for oxidized low-density lipoprotein and scavenger receptors SR-AI, CD36, and LOX-1 in aortic valve stenosis. Atherosclerosis.

[29] Skenteris N.T., Seime T., Witasp A., Karlöf E., Wasilewski G.B., Heuschkel M.A., Jaminon A.M.G., Oduor L., Dzhanaev R., Kronqvist M. (2022). Osteomodulin attenuates smooth muscle cell osteogenic transition in vascular calcification. Clin. Transl. Med..

[30] Maeda K., Ma X., Chalajour F., Hanley F.L., Riemer R.K. (2016). Critical role of coaptive strain in aortic valve leaflet homeostasis: Use of a novel flow culture bioreactor to explore heart valve mechanobiology. J. Am. Heart Assoc..

[31] Reininger A.J. (2008). VWF attributes–impact on thrombus formation. Thromb. Res..

[32] Thériault S., Dina C., Messika-Zeitoun D., Le Scouarnec S., Capoulade R., Gaudreault N., Rigade S., Li Z., Simonet F., Lamontagne M. (2019). Genetic association analyses highlight IL6, ALPL, and NAV1 as 3 new susceptibility genes underlying calcific aortic valve stenosis. Circ. Genom. Precis. Med..

[33] Ghazvini-Boroujerdi M., Clark J., Narula N., Palmatory E., Connolly J.M., DeFelice S., Xu J., Jian B., Hazelwood S., Levy R.J. (2004). Transcription factor Egr-1 in calcific aortic valve disease. J. Heart Valve Dis..

[34] Wang M., Hao H., Leeper N.J., Zhu L. (2018). Thrombotic regulation from the endothelial cell perspectives. Arterioscler. Thromb. Vasc. Biol..

[35] Jeong D., Lee M.A., Li Y., Yang D.K., Kho C., Oh J.G., Hong G., Lee A., Song M.H., LaRocca T.J. (2016). Matricellular protein CCN5 reverses established cardiac fibrosis. J. Am. Coll. Cardiol..

[36] Posey K.L., Hecht J.T. (2008). The role of cartilage oligomeric matrix protein (COMP) in skeletal disease. Curr. Drug Targets.

[37] Mao D., Epple H., Uthgenannt B., Novack D.V., Faccio R. (2006). PLCgamma2 regulates osteoclastogenesis via its interaction with ITAM proteins and GAB2. J. Clin. Investig..

[38] Takalo M., Wittrahm R., Wefers B., Parhizkar S., Jokivarsi K., Kuulasmaa T., Mäkinen P., Martiskainen H., Wurst W., Xiang X. (2020). The Alzheimer’s disease-associated protective Plcγ2-P522R variant promotes immune functions. Mol. Neurodegener..

[39] Tsai A.P., Dong C., Lin P.B., Messenger E.J., Casali B.T., Moutinho M., Liu Y., Oblak A.L., Lamb B.T., Landreth G.E. (2022). PLCG2 is associated with the inflammatory response and is induced by amyloid plaques in Alzheimer’s disease. Genome Med..

[40] Li G., Qiao W., Zhang W., Li F., Shi J., Dong N. (2017). The shift of macrophages toward M1 phenotype promotes aortic valvular calcification. J. Thorac. Cardiovasc. Surg..

[41] Zernecke A., Winkels H., Cochain C., Williams J.W., Wolf D., Soehnlein O., Robbins C.S., Monaco C., Park I., McNamara C.A. (2020). Meta-analysis of leukocyte diversity in atherosclerotic mouse aortas. Circ. Res..

[42] Jarrett M.J., Houk A.K., McCuistion P.E., Weyant M.J., Reece T.B., Meng X., Fullerton D.A. (2021). Wnt signaling mediates pro-fibrogenic activity in human aortic valve interstitial cells. Ann. Thorac. Surg..

[43] Odiete O., Hill M.F., Sawyer D.B. (2012). Neuregulin in cardiovascular development and disease. Circ. Res..

[44] Chen T., Wang Y., Hao Z., Hu Y., Li J. (2021). Parathyroid hormone and its related peptides in bone metabolism. Biochem. Pharmacol..

[45] Mahajan G.J., Vallender E.J., Garrett M.R., Challagundla L., Overholser J.C., Jurjus G., Dieter L., Syed M., Romero D.G., Benghuzzi H. (2018). Altered neuro-inflammatory gene expression in hippocampus in major depressive disorder. Prog. Neuropsychopharmacol. Biol. Psychiatry.

