# Supplementary material for: Side- and Disease-Dependent Changes in Human Aortic Valve Cell Population and Transcriptomic Heterogeneity Determined by Single-Cell RNA Sequencing
Source: Genes (Basel). 2024 Dec 19;15(12):1623. doi: 10.3390/genes15121623 (PMC11675841; doi:10.3390/genes15121623)
Supplement: Supplementary file 1 [file genes-15-01623-s001.zip › genes-3356920-supplementary-update.pdf]

Supplementary Material

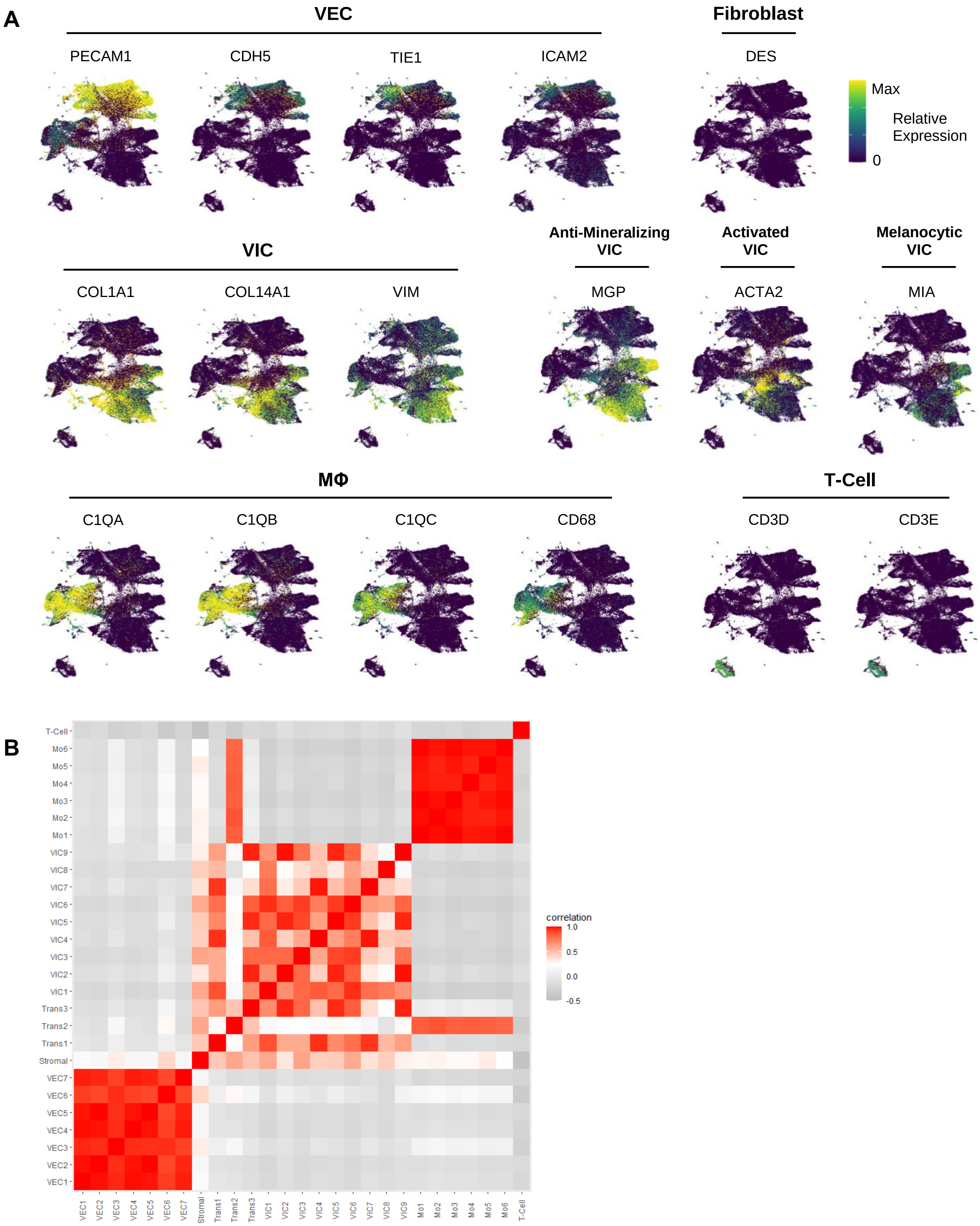

Supplementary Figure S1. Annotation markers for cell clustering. A. Feature plots of annotation markers for labeling cell lineages. **B.** Correlation map denoting average expressions of all annotation markers between cell clusters.

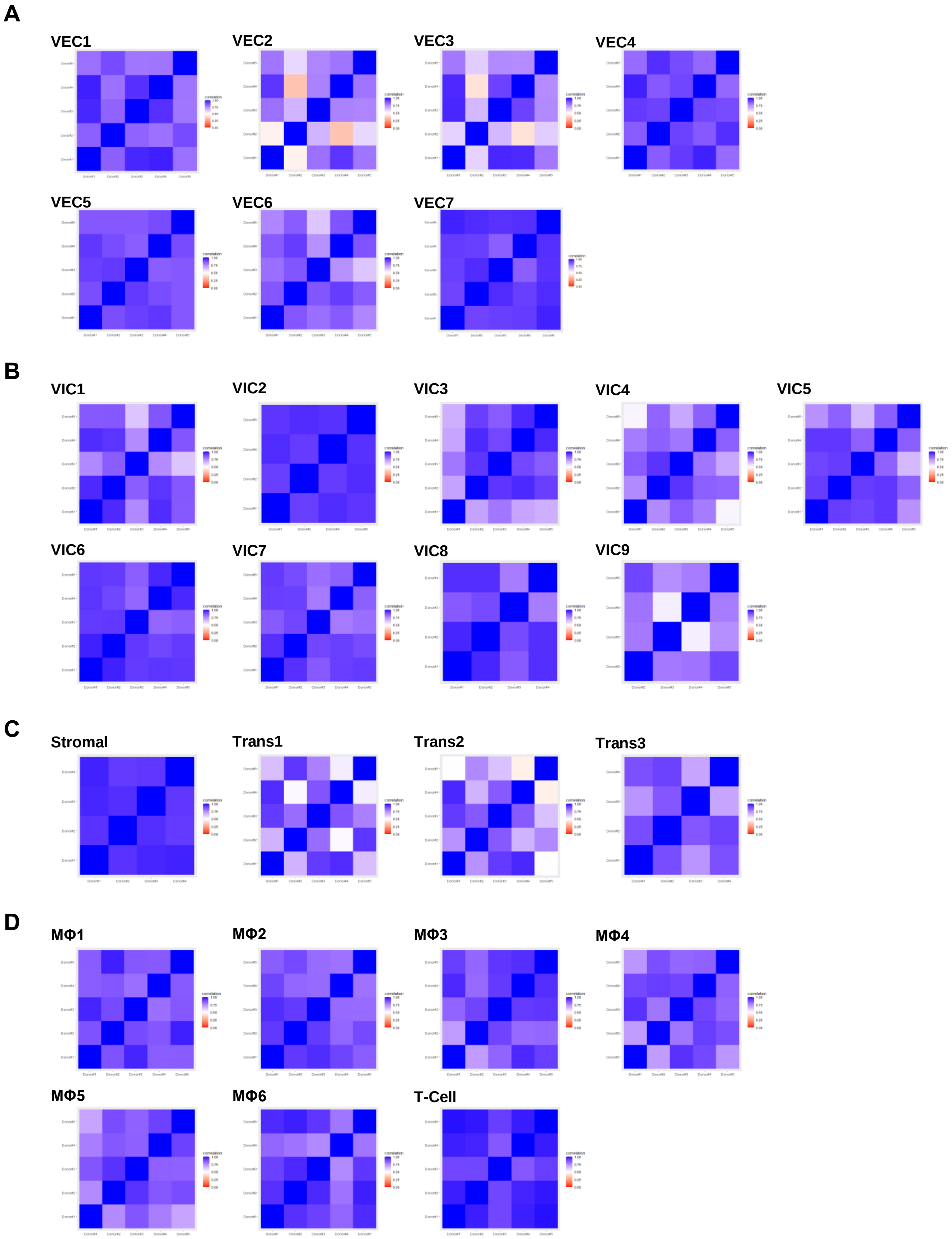

A
VEC1
VEC2
VEC3
VEC4
VEC5
VEC6
VEC7
B
VIC1
VIC2
VIC3
VIC4
VIC5
VIC6
VIC7
VIC8
VIC9
C
Stromal
Trans1
Trans2
Trans3
D
MΦ1
MΦ2
MΦ3
MΦ4
MΦ5
MΦ6
T-Cell

Supplementary Figure S2. No significant batch effect of single-cell RNA sequencing found between donors. A-D. Correlation maps denoting average expressions of all genes between donors for individual clusters of valvular endothelial cells (**A**), valvular interstitial cells (**B**), transitional cells (**C**), and immune cells (**D**).

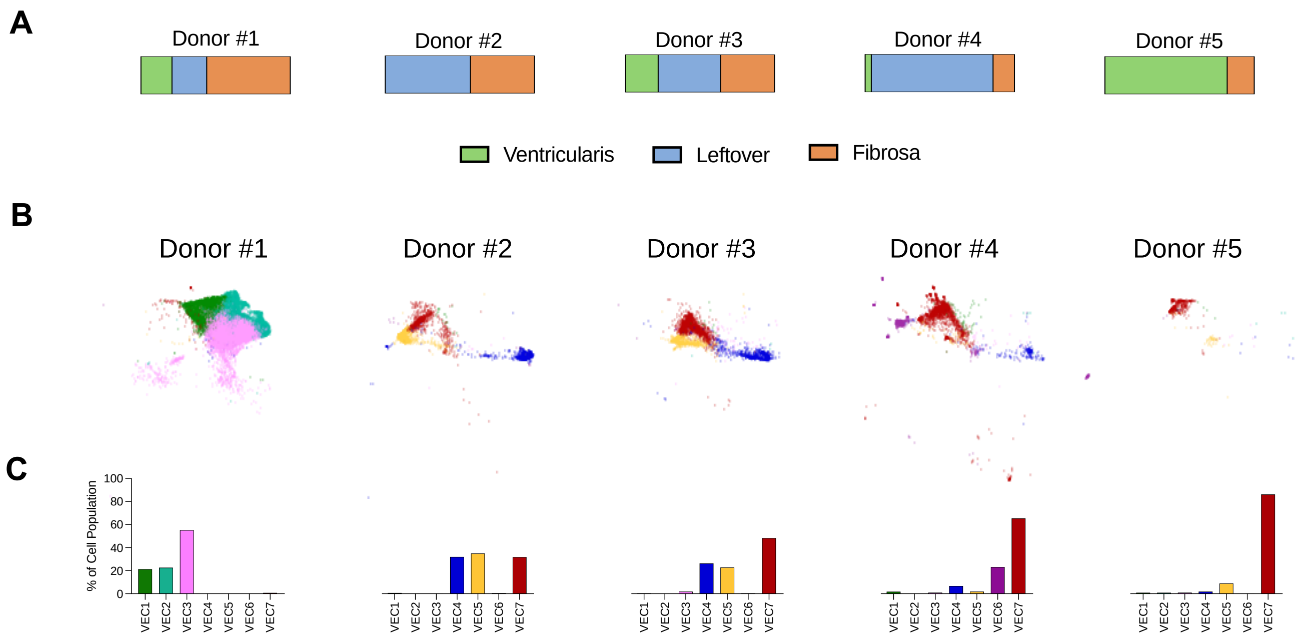

Supplementary Figure S3. VEC clustering patterns in each donor. A. Total sample proportions in individual donors. **B.** UMAP of the VEC separated by donor. **C.** Quantification of % VEC population in each donor. **D.** Quantification of fibrosa (solid) or ventricularis (striped) proportion of individual VEC clusters in each donor.

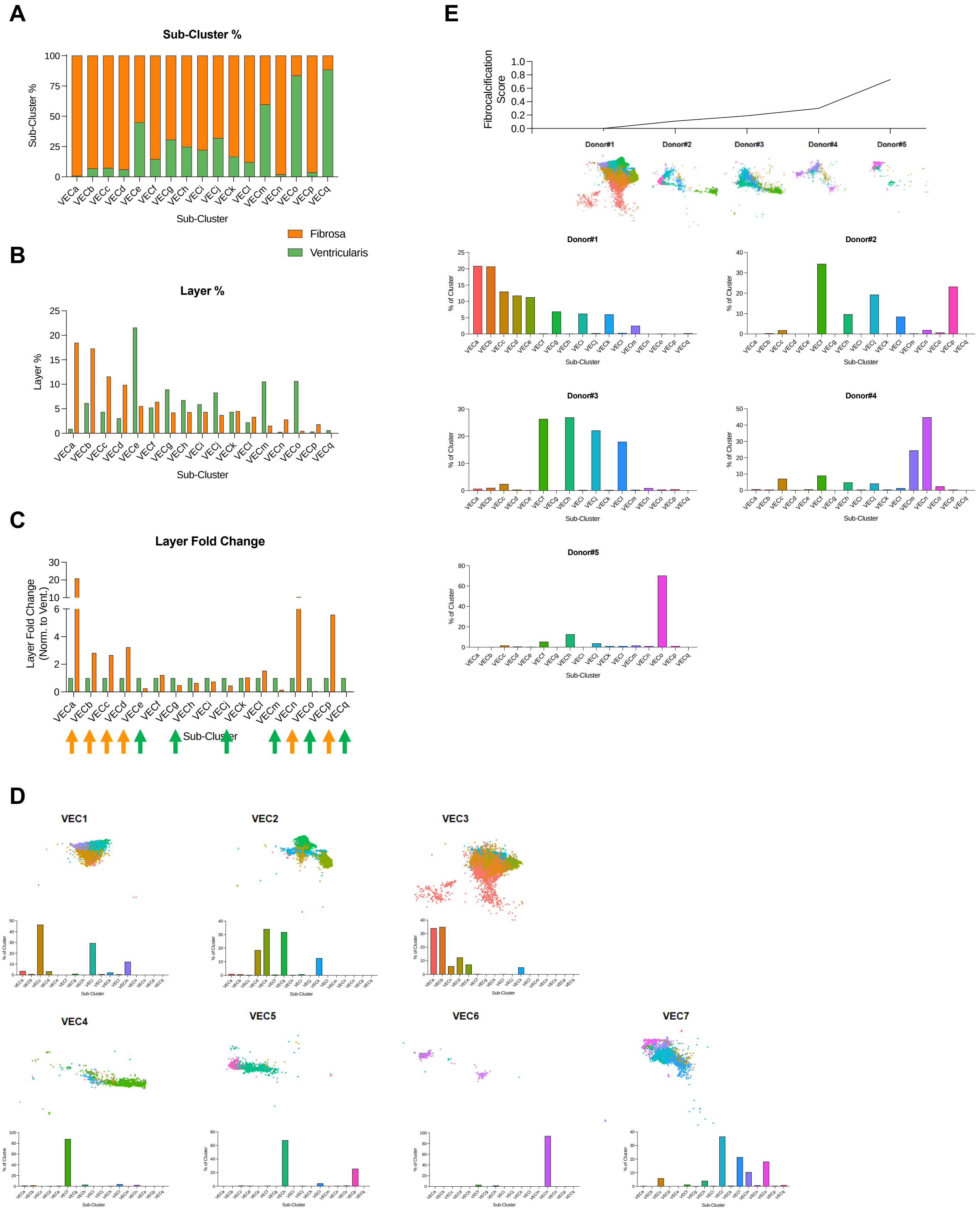
A
Sub-Cluster %
Sub-Cluster %
Sub-Cluster
Fibrosa
Ventricularis
B
Layer %
Layer %
Sub-Cluster
C
Layer Fold Change
Layer Fold Change (Norm. to Vent.)
Sub-Cluster
D
VEC1
VEC2
VEC3
VEC4
VEC5
VEC6
VEC7
E
Fibrocalcification Score
Donor#1
Donor#2
Donor#3
Donor#4
Donor#5
% of Cluster
Sub-Cluster
VECa
VECb
VECc
VECd
VECe
VECf
VECg
VECh
VECi
VECj
VECk
VECl
VECm
VECn
VECo
VECp
VECq

Supplementary Figure S4. Sub-clustering analysis of VEC. A. Breakdown of VEC sub-cluster by layer of origin reported as sub-cluster % (orange = fibrosa VEC, green = ventricularis VEC). **B.** Layer VEC sub-cluster proportional composition reported as layer %. **C.** Layer VEC sub-cluster proportional composition normalized to ventricularis reported as layer fold change (orange arrows = fibrosa layer fold change > 2 compared to ventricularis, green arrows = fibrosa layer fold change < 0.5 compared to ventricularis). **D.** UMAP of VEC sub-clusters separated by original VEC clusters and quantification of sub-cluster proportional composition. **E.** UMAP of VEC sub-clusters separated by fibrocalcification score and quantification of sub-cluster proportional composition of donor VEC subset.

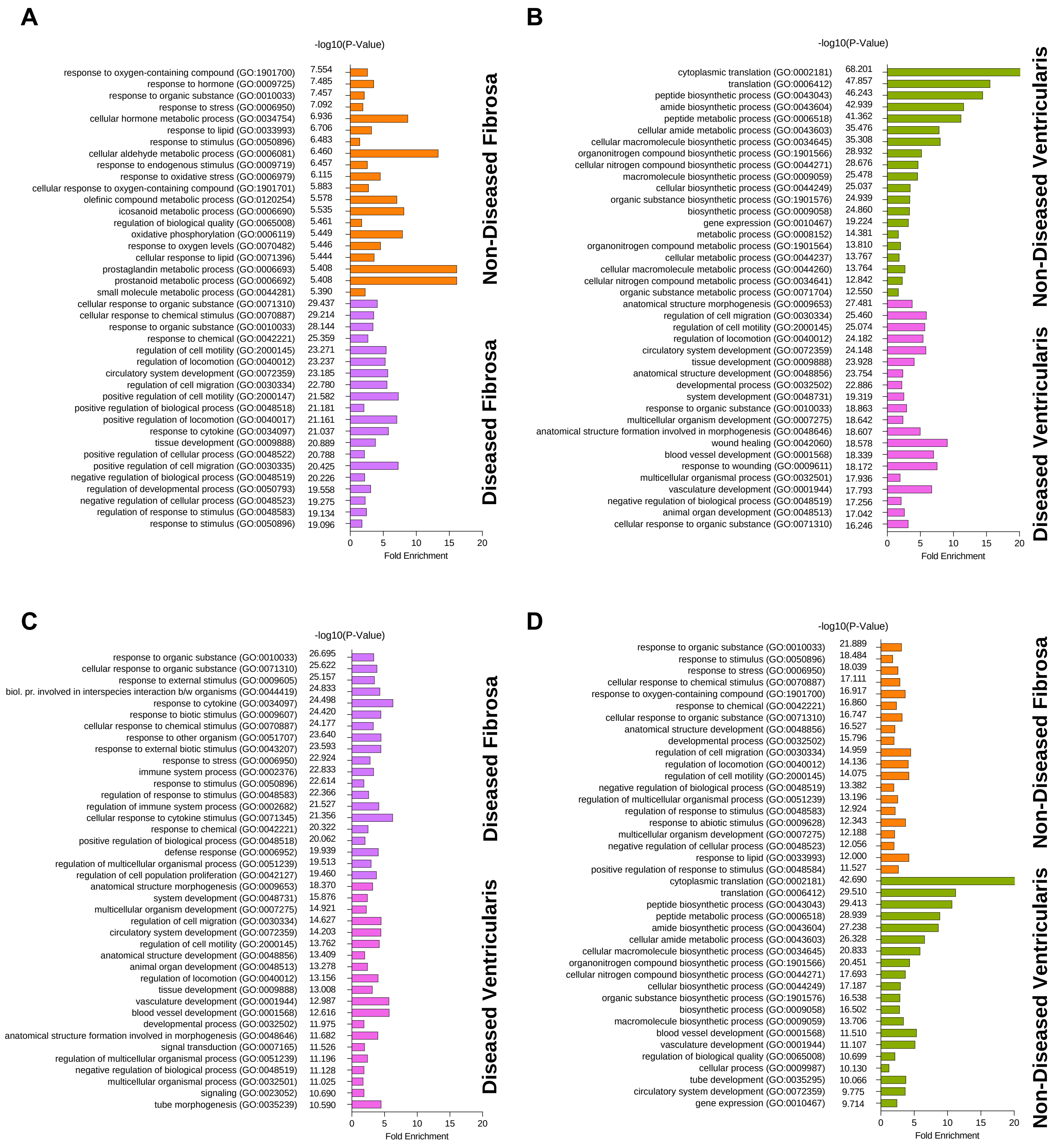

Supplementary Figure S5. Gene ontology analysis of side- and disease stage-dependent sub-clusters. A-D. Top 20 significantly enriched biological processes resulting from a gene ontology analysis using the top 200 upregulated genes in each VEC sub-cluster resulting from disease stage-dependent pairwise comparisons (non-diseased fibrosa vs. diseased fibrosa [**A**] or non-diseased ventricularis vs. diseased ventricularis [**B**]) or side-dependent pairwise comparisons (diseased fibrosa vs. diseased ventricularis [**C**] or non-diseased fibrosa vs. non-diseased ventricularis [**D**]).

A

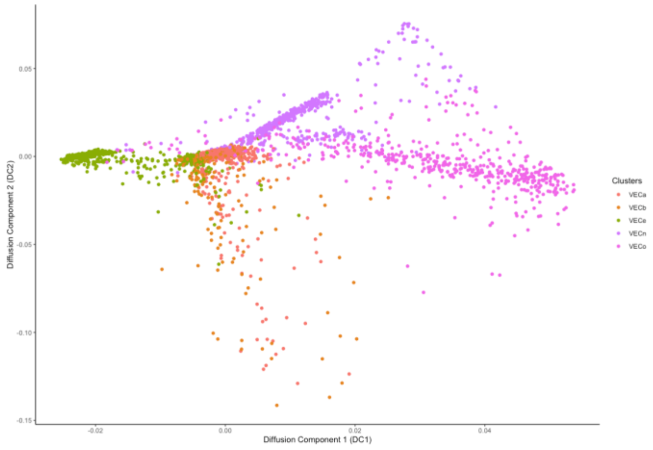

B

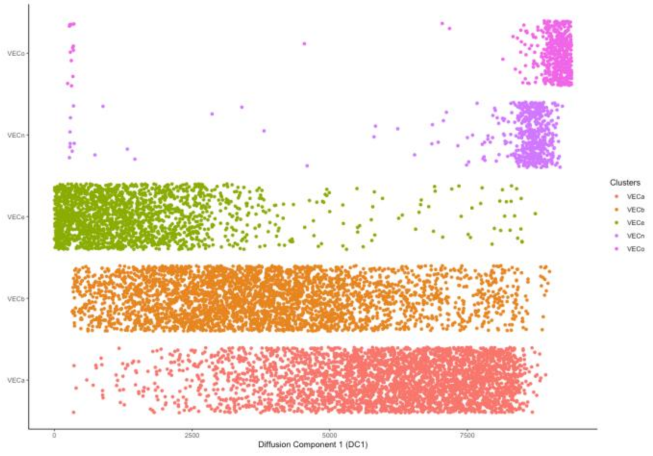

Supplementary Figure S6. Diffusion map and pseudotime analysis of side- and disease stage-dependent VEC sub-clusters. A. Diffusion map of VEC sub-clusters: non-diseased fibrosa = VEC_a (red) and VEC_b (orange), non-diseased ventricularis = VEC_e (green), diseased fibrosa = VEC_n (violet), and diseased ventricularis = VEC_o (pink). **B.** Pseudotime analysis of side- and disease stage-dependent VEC sub-clusters ordered by Diffusion Component 1 (DC1).

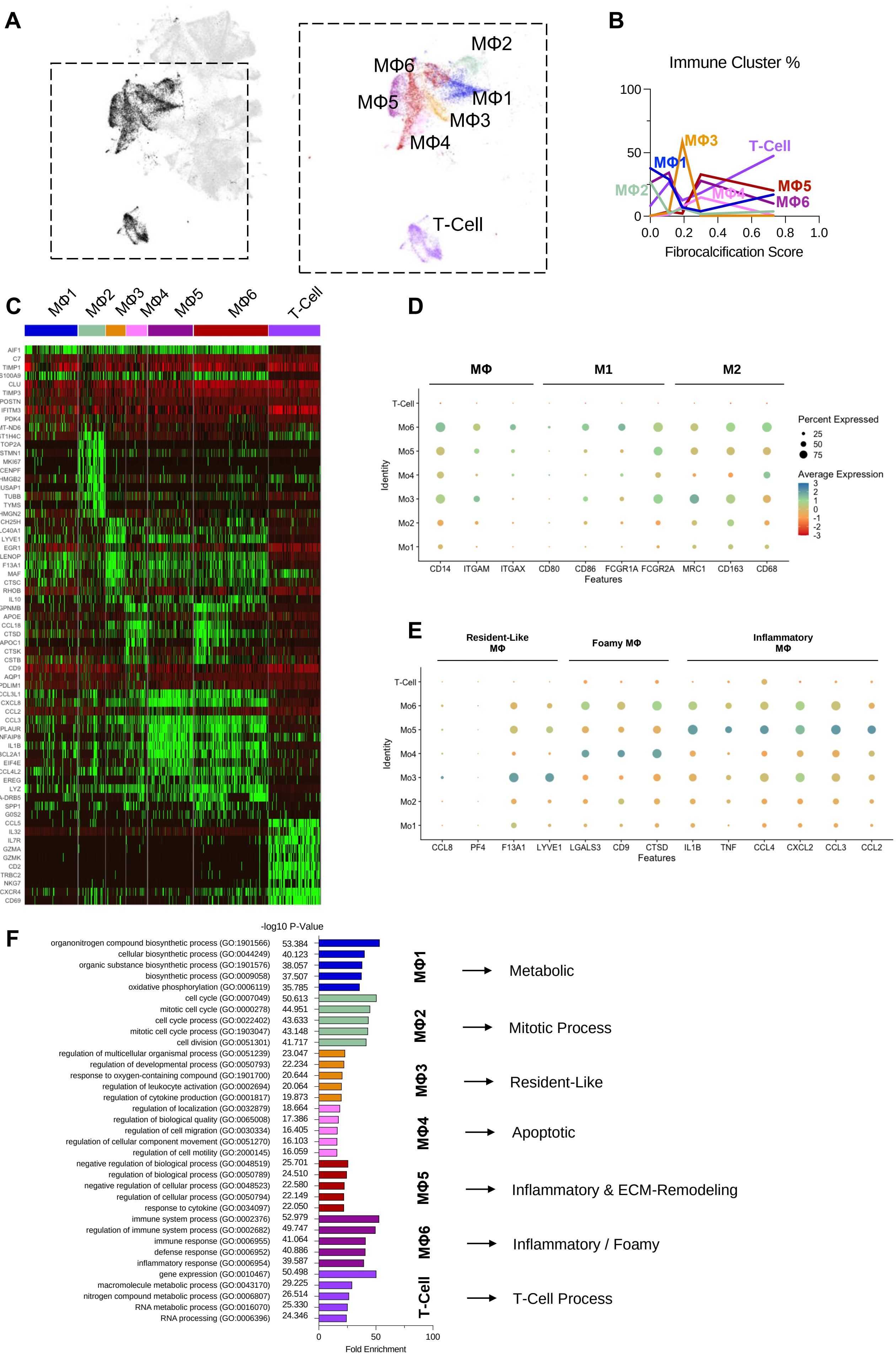
A
MΦ2
MΦ6
MΦ5
MΦ1
MΦ3
MΦ4
T-Cell
B
Immune Cluster %
100
50
0
MΦ3
T-Cell
MΦ1
MΦ2
MΦ4
MΦ5
MΦ6
0.0 0.2 0.4 0.6 0.8 1.0
Fibrocalcification Score
C
MΦ1 MΦ2 MΦ3 MΦ4 MΦ5 MΦ6 T-Cell
AIF1
C7
TIMP1
S100A9
CLU
TIMP3
POSTN
IFITM3
PDK4
MT-ND6
HIST1H4C
TOP2A
STMN1
MKI67
CENPF
HMGB2
NUSAP1
TUBB
TYMS
HMGN2
CH25H
SLC40A1
LYVE1
EGR1
SELENOP
F13A1
MAF
CTSC
RHOB
IL10
GPNMB
APOE
CCL18
CTSD
APOC1
CTSK
CSTB
CD9
AQP1
PDLIM1
CCL3L1
CXCL8
CCL2
CCL3
PLAUR
TNFAIP8
IL1B
BCL2A1
EIF4E
CCL4L2
EREG
LYZ
HLA-DRB5
SPP1
G0S2
CCL5
IL32
IL7R
GZMA
GZMK
CD2
TRBC2
NKG7
CXCR4
CD69
D
MΦ
M1
M2
T-Cell
Mo6
Mo5
Mo4
Mo3
Mo2
Mo1
Identity
CD14 ITGAM ITGAX CD80 CD86 FCGR1A FCGR2A MRC1 CD163 CD68
Features
Percent Expressed
25
50
75
Average Expression
3
2
1
0
-1
-2
-3
E
Resident-Like MΦ
Foamy MΦ
Inflammatory MΦ
T-Cell
Mo6
Mo5
Mo4
Mo3
Mo2
Mo1
Identity
CCL8 PF4 F13A1 LYVE1 LGALS3 CD9 CTSD IL1B TNF CCL4 CXCL2 CCL3 CCL2
Features
F
-log10 P-Value
organonitrogen compound biosynthetic process (GO:1901566) 53.384
cellular biosynthetic process (GO:0044249) 40.123
organic substance biosynthetic process (GO:1901576) 38.057
biosynthetic process (GO:0009058) 37.507
oxidative phosphorylation (GO:0006119) 35.785
cell cycle (GO:0007049) 50.613
mitotic cell cycle (GO:0000278) 44.951
cell cycle process (GO:0022402) 43.633
mitotic cell cycle process (GO:1903047) 43.148
cell division (GO:0051301) 41.717
regulation of multicellular organismal process (GO:0051239) 23.047
regulation of developmental process (GO:0050793) 22.234
response to oxygen-containing compound (GO:1901700) 20.644
regulation of leukocyte activation (GO:0002694) 20.064
regulation of cytokine production (GO:0001817) 19.873
regulation of localization (GO:0032879) 18.664
regulation of biological quality (GO:0065008) 17.386
regulation of cell migration (GO:0030334) 16.405
regulation of cellular component movement (GO:0051270) 16.103
regulation of cell motility (GO:2000145) 16.059
negative regulation of biological process (GO:0048519) 25.701
regulation of biological process (GO:0050789) 24.510
negative regulation of cellular process (GO:0048523) 22.580
regulation of cellular process (GO:0050794) 22.149
response to cytokine (GO:0034097) 22.050
immune system process (GO:0002376) 52.979
regulation of immune system process (GO:0002682) 49.747
immune response (GO:0006955) 41.064
defense response (GO:0006952) 40.886
inflammatory response (GO:0006954) 39.587
gene expression (GO:0010467) 50.498
macromolecule metabolic process (GO:0043170) 29.225
nitrogen compound metabolic process (GO:0006807) 26.514
RNA metabolic process (GO:0016070) 25.330
RNA processing (GO:0006396) 24.346
0 50 100
Fold Enrichment
MΦ1 → Metabolic
MΦ2 → Mitotic Process
MΦ3 → Resident-Like
MΦ4 → Apoptotic
MΦ5 → Inflammatory & ECM-Remodeling
MΦ6 → Inflammatory / Foamy
T-Cell → T-Cell Process

Supplementary Figure S7. Immune cell clustering patterns change with disease stage. A. UMAP of the 6 MΦ clusters and 1 T-Cell cluster. **B.** Immune cell cluster proportions change as a function of disease stage. **C.** Heatmap highlighting top 10 most enriched genes in each immune cell cluster. **D.** Canonical MΦ (*CD14*, *ITGAM*, and *ITGAX*), M1 MΦ (*CD80*, *CD86*, *FCGR1A*, and *FCGR2A*), and M2 MΦ (*MRC1*, *CD163*, and *CD68*) marker expression in each immune cell cluster. **E.** Resident-like MΦ (*CCL8*, *PF4*, *F13A1*, and *LYVE1*), Foamy MΦ (*LGALS3*, *CD9*, and *CTSD*), and Inflammatory MΦ (*IL1B*, *TNF*, *CCL4*, *CXCL2*, *CCL3*, and *CCL2*) marker expression, as reported by *Zernecke et al 2020*, in each immune cell cluster. **F.** Top 5 significantly enriched biological processes from a gene ontology analysis using the top 200 upregulated genes in each immune cell cluster and a representative process for each cluster.

MΦ: macrophage

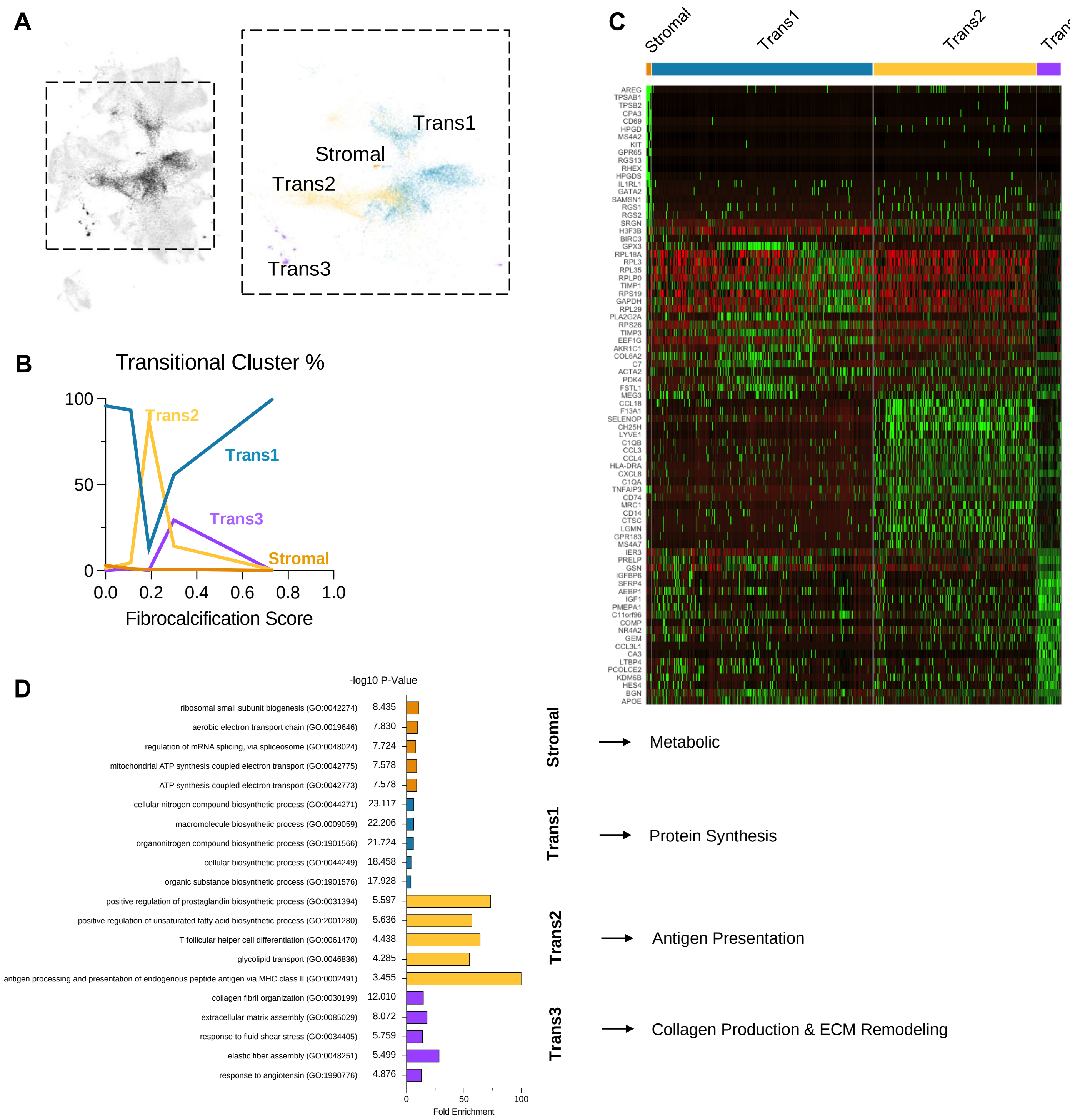

Supplementary Figure S8. Transitional cell clustering patterns change with disease stage. A. UMAP of the 3 transitional cell clusters and 1 Stromal cluster. **B.** Transitional cell cluster proportions change as a function of disease stage. **C.** Heatmap highlighting top 10 most enriched genes in each transitional cell cluster. **D.** Top 5 significantly enriched biological processes from a gene ontology analysis using the top 200 upregulated genes in each transitional cell cluster and a representative process for each cluster.

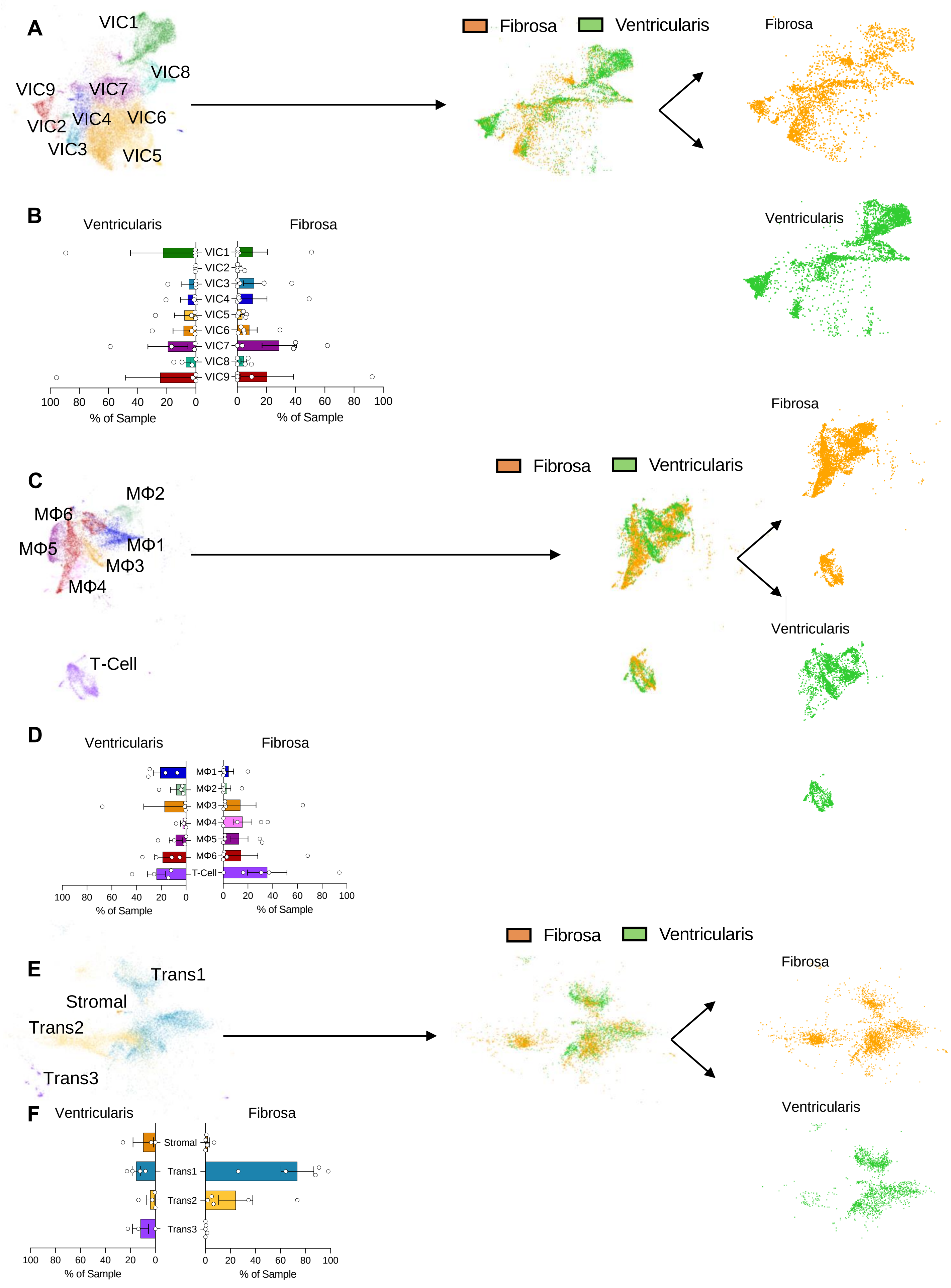

A
VIC1
VIC8
VIC9
VIC7
VIC2
VIC4
VIC6
VIC3
VIC5
Fibrosa
Ventricularis
Fibrosa
Ventricularis
B
Ventricularis
Fibrosa
VIC1
VIC2
VIC3
VIC4
VIC5
VIC6
VIC7
VIC8
VIC9
100 80 60 40 20 0
0 20 40 60 80 100
% of Sample
% of Sample
C
MΦ2
MΦ6
MΦ5
MΦ1
MΦ3
MΦ4
T-Cell
Fibrosa
Ventricularis
Fibrosa
Ventricularis
D
Ventricularis
Fibrosa
MΦ1
MΦ2
MΦ3
MΦ4
MΦ5
MΦ6
T-Cell
100 80 60 40 20 0
0 20 40 60 80 100
% of Sample
% of Sample
E
Trans1
Stromal
Trans2
Trans3
Fibrosa
Ventricularis
Fibrosa
Ventricularis
F
Ventricularis
Fibrosa
Stromal
Trans1
Trans2
Trans3
100 80 60 40 20 0
0 20 40 60 80 100
% of Sample
% of Sample

Supplementary Figure S9. Side-dependent clustering analysis of VIC, immune, and transitional cells. A. Side-dependent separation of VEC UMAP into fibrosa vs ventricularis. **B.** VIC cluster proportions in ventricularis vs fibrosa. **C.** Side-dependent separation of immune cell UMAP into fibrosa vs ventricularis. **D.** Immune cell cluster proportions in ventricularis vs fibrosa. **E.** Side-dependent separation of transitional cell UMAP into fibrosa vs ventricularis. **F.** Transitional cell cluster proportions in ventricularis vs fibrosa.

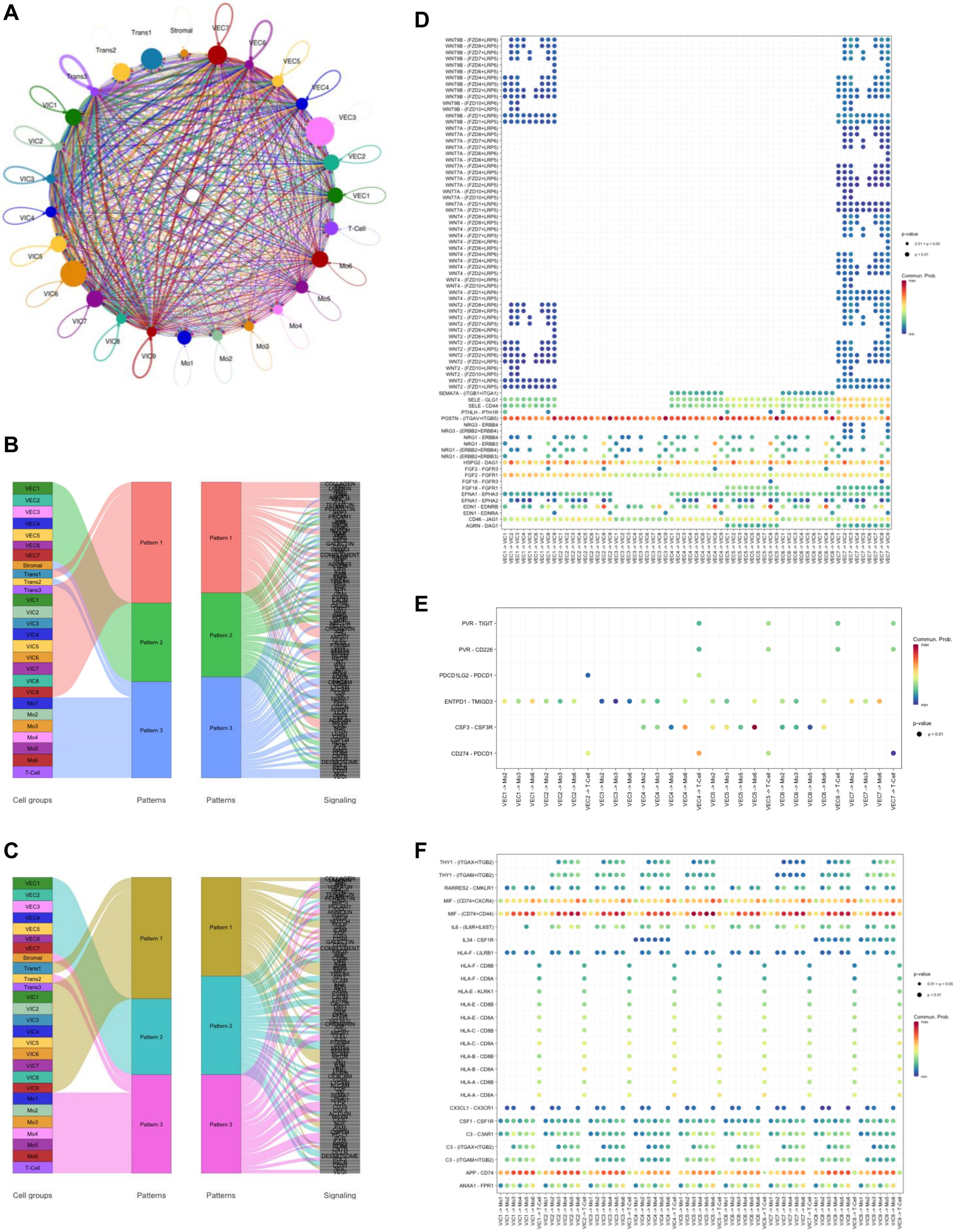
A
Trans1
Stromal
VEC7
VEC6
VEC5
VEC4
VEC3
VEC2
VEC1
T-Cell
Mo6
Mo5
Mo4
Mo3
Mo2
Mo1
VIC9
VIC8
VIC7
VIC6
VIC5
VIC4
VIC3
VIC2
VIC1
Trans3
Trans2
B
Pattern 1
Pattern 2
Pattern 3
Cell groups
Patterns
Patterns
Signaling
C
Pattern 1
Pattern 2
Pattern 3
Cell groups
Patterns
Patterns
Signaling
D
p-value
Commun. Prob.
E
PVR - TIGIT
PVR - CD226
PDCD1LG2 - PDCD1
ENTPD1 - TMIGD3
CSF3 - CSF3R
CD274 - PDCD1
Commun. Prob.
p-value
F
THY1 - (ITGAX+ITGB2)
THY1 - (ITGAM+ITGB2)
RARRES2 - CMKLR1
MIF - (CD74+CXCR4)
MIF - (CD74+CD44)
IL6 - (IL6R+IL6ST)
IL34 - CSF1R
HLA-F - LILRB1
HLA-F - CD8B
HLA-F - CD8A
HLA-E - KLRK1
HLA-E - CD8B
HLA-E - CD8A
HLA-C - CD8B
HLA-C - CD8A
HLA-B - CD8B
HLA-B - CD8A
HLA-A - CD8B
HLA-A - CD8A
CX3CL1 - CX3CR1
CSF1 - CSF1R
C3 - C3AR1
C3 - (ITGAX+ITGB2)
C3 - (ITGAM+ITGB2)
APP - CD74
ANXA1 - FPR1
p-value
Commun. Prob.

Supplementary Figure S10. Predictive cell-cell interaction analyses with CellChat. A. Communication network chord map depicting all cell-cell interactions predicted from the scRNAseq dataset. **B.** Alluvial plot showing all outgoing communication patterns from individual cell clusters. **C.** Alluvial plot showing all incoming communication patterns to individual cell clusters. **D-F.** Significantly enriched ($p < 0.05$, minimum communication probability = blue, maximum communication probability = red) ligand-receptor interactions between VEC and VIC (**D**), VEC and immune cells (**E**), and VIC and immune cells (**F**).

A

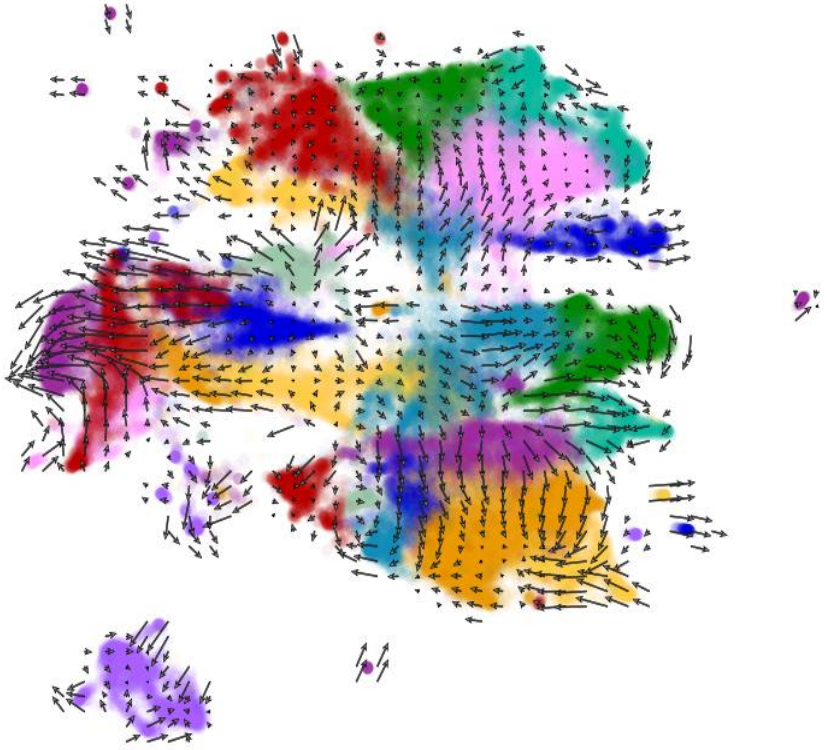

B

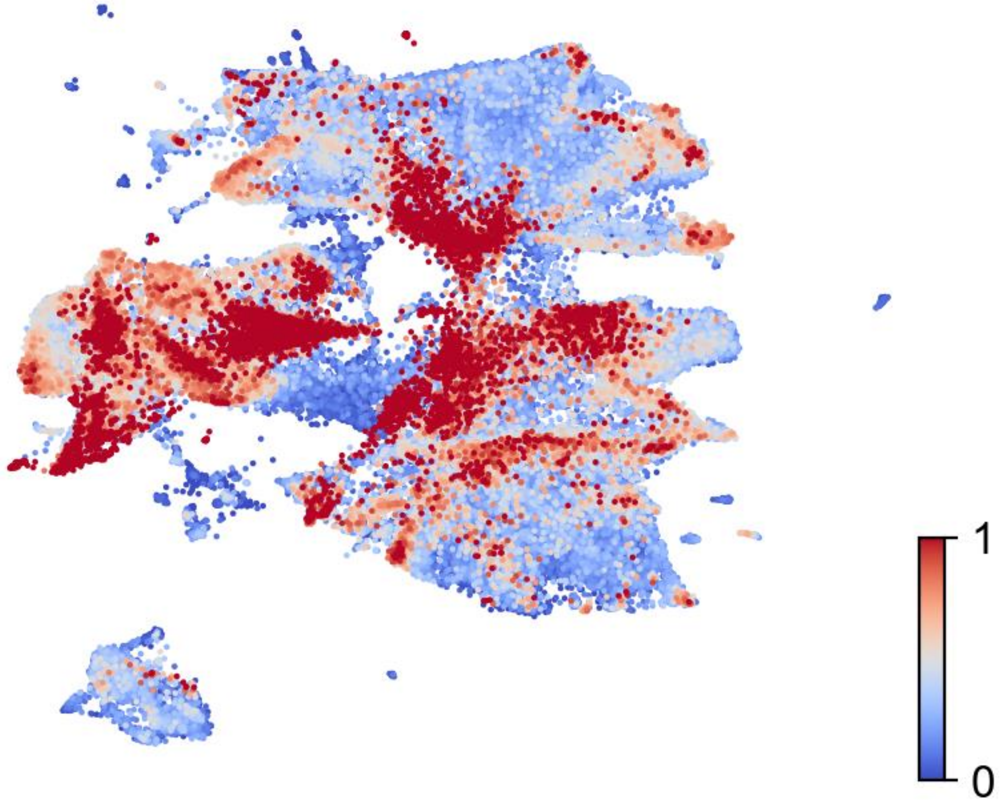

Supplementary Figure S11. RNA Velocity analysis to predict cell cluster transitions. A. Velocity vector map overlayed onto UMAP to show cluster transitions. **B.** Normalized velocity length (0 = Blue, 1 = Red) for individual cells in dataset.

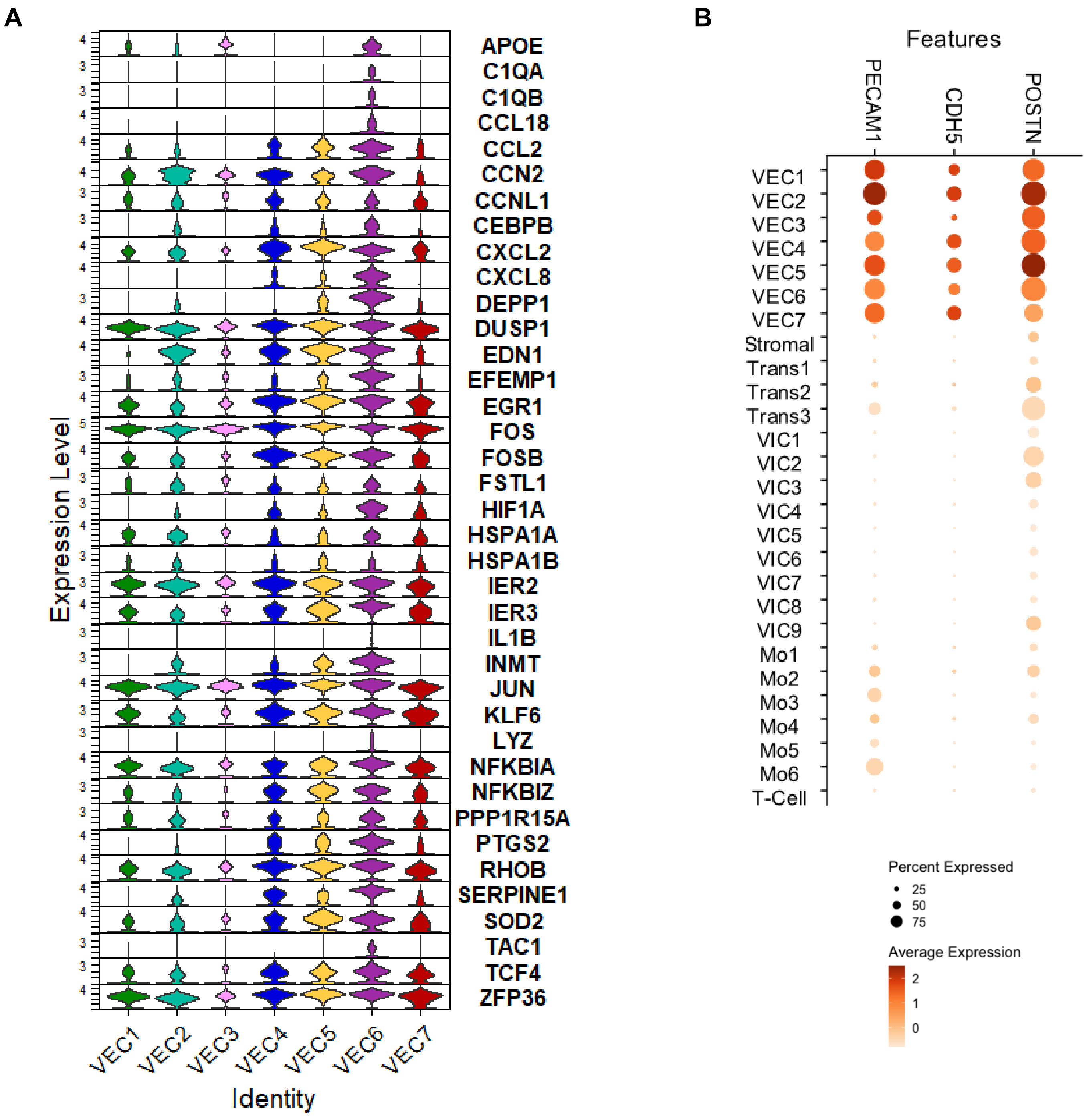

Supplementary Figure S12. Side-dependent genes upregulated in the VEC fibrosa are predominantly increased in disease-associated VEC clusters (VEC4-7). A. Stacked violin plot comparing the expression of the identified potential therapeutic targets (upregulated in diseased fibrosa subsets in scRNAseq) across the original VEC clusters from the merged dataset. **B.** Dot plot showing average expression levels of *PECAM1*, *CDH5*, and *POSTN* across all identified cell clusters.
